# Socioeconomic inequalities in early adulthood disrupt the immune transcriptomic landscape via upstream regulators

**DOI:** 10.1038/s41598-024-51517-6

**Published:** 2024-01-13

**Authors:** Sudharshan Ravi, Michael J. Shanahan, Brandt Levitt, Kathleen Mullan Harris, Steven W. Cole

**Affiliations:** 1 Jacobs Center for Productive Youth Development, University of Zürich, Andreasstrasse 15, 8050 Zürich, Switzerland; 2 Department of Sociology, University of Zürich, 8050 Zürich, Switzerland; 3 Carolina Population Center, University of North Carolina at Chapel Hill, Chapel Hill, NC 27516 USA; 4 Department of Sociology, University of North Carolina at Chapel Hill, Chapel Hill, NC 27599-3210 USA; 5 School of Medicine, University of California, Los Angeles, CA 90095 USA

**Keywords:** Computational biology and bioinformatics, Health care, Risk factors

## Abstract

Disparities in socio-economic status (SES) predict many immune system-related diseases, and previous research documents relationships between SES and the immune cell transcriptome. Drawing on a bioinformatically-informed network approach, we situate these findings in a broader molecular framework by examining the upstream regulators of SES-associated transcriptional alterations. Data come from the National Longitudinal Study of Adolescent to Adult Health (Add Health), a nationally representative sample of 4543 adults in the United States. Results reveal a network—of differentially expressed genes, transcription factors, and protein neighbors of transcription factors—that shows widespread SES-related dysregulation of the immune system. Mediational models suggest that body mass index (BMI) plays a key role in accounting for many of these associations. Overall, the results reveal the central role of upstream regulators in socioeconomic differences in the molecular basis of immunity, which propagate to increase risk of chronic health conditions in later-life.

## Introduction

A considerable body of evidence suggests that disparities in SES—reflecting education, income, occupational prestige, and subjective status—play a critical role in shaping the health trajectories of people, with lower SES associated with elevated morbidity and mortality rates^1–6^. Many of these diseases—including, for example, asthma^7^, atopic dermatitis^8^, food allergies^9^, systemic lupus erythematosus^10^, and periodontal disease^11^—vary widely in their pathologies but share a common etiological pathway involving immune dysregulation, and they are more common in lower socioeconomic strata than among people with higher SES^12^. Three strands of evidence also document associations between SES and biomarkers of the immune system. First, many studies report associations between childhood SES and pro-inflammatory markers in circulating peripheral blood (such as interleukin-6 and C-reactive protein (CRP)^13^) that, if chronically activated, presage a wide-range of diseases including, for example, diabetes type 2, some cancers, and cardiovascular disease. Second, studies have also examined white blood cell composition, finding that low SES is associated with increased development and circulation of pro-inflammatory immune cells (monocytes and neutrophils^14^), whereas parental education is positively associated with the proportion of lymphocytes and negatively associated with the proportion of neutrophils and, among older adults, that SES is related to shifts in cell composition indicative of immunosenescence^15–18^. And finally, a limited number of studies have examined functional assays of immune response, sometimes ex-vivo, and show that, once again, childhood socioeconomic status is a risk factor for immune dysregulation, possibly more so among boys^19–22^. Nevertheless, despite the abundance of evidence connecting socioeconomic inequalities to immune-related diseases and biomarkers, the molecular etiology of SES-mediated immune alterations remains less explored.

A growing number of studies have examined SES and transcriptional patterns indicative of immune functioning. Research consistently shows people from low SES backgrounds have greater proinflammatory activity^14,22–26^. Additionally, SES is associated with the expression of genes regulated by the glucocorticoid receptor and interferon response factors, suggesting a suppression of adaptive immunity and innate antiviral immunity^22,27^. This signature pattern—involving the upregulation of proinflammatory genes and the downregulation of type I interferon innate antiviral response genes among the lower strata of status, called the “Conserved Transcriptional Response to Adversity” (CTRA)—has been observed in numerous populations with a range of research designs. Essentially, SES is associated with CTRA activation, which in turn is associated with the molecular underpinnings of immune-related and inflammatory diseases^22,28–30^.

The current study seeks to expand on these findings by providing a broader mapping of associations between SES and the molecular signaling pathways that regulate immunity. Understanding the impact of socioeconomic status on immune gene expression requires a systems-oriented approach that extends beyond the functional examination of individually differentially expressed genes and includes the network of upstream regulators. Furthermore, traditional transcription factor binding motif enrichment analysis suffers from the lack of tissue specificity. We aim to address these drawbacks in this paper by detailing upstream tissue-specific transcriptional factors and protein-protein interactors as a networked system, along with differentially regulated genes, that responds to SES. Such an approach offers a comprehensive understanding of how SES is associated with the molecular mechanisms that drive biological processes such as gene expression, cell signaling, and cell fate, which ultimately lead to disease in individuals of lower SES^31,32^. Significantly, we leverage the comprehensive resource of human tissue-specific gene regulatory network of Marbach et al.^33^ to isolate transcription factors that could play a pivotal role in modulating the expression of SES—differentially expressed genes in whole blood. Additionally, by incorporating direct protein-protein interactors, we create a broad, inclusive and holistic set of genes and proteins that act as a networked system in disrupting essential biological processes. Our approach reveals the decisive role of transcription factors in driving SES—associated dysregulation that previously would be attributed to SES—differentially expressed genes.

Emerging evidence points to the intricate interplay between low socioeconomic position and high body mass index (BMI)^34,35^. Recent meta-analyses have consistently linked lowered socioeconomic status and elevated inflammatory biomarkers largely via obesity^36,37^. Although BMI is an imperfect indicator of obesity as it does not distinguish fat from fat-free mass, Liu et al.^38^ observed a likely strong mediation of BMI in the negative relationship between childhood SES and adulthood inflammatory marker C reactive protein (CRP). Additionally, previous systematic reviews have shown that improved indicators of fat and obesity follow a similar pattern of health disparity to those seen with BMI^37^. Thus, we examine the potential mediation of BMI, as an indicator of obesity, along with other common social and behavioral mediators of SES, and the entire system of differentially expressed genes, upstream transcription factors and protein-protein interactors that drive immune dysregulation as a result of lowered SES.

We focus on American adults in their late 1930s, who are ostensibly healthy but nevertheless at-risk for later health challenges. We leverage the mRNA data from 4543 early adults participating in the National Longitudinal Study of Adolescent Health (Add Health)^39^. First, we identify cell functional pathways and their directionality in SES-related dysregulation of the immune system. To this end, we capitalize on publicly available pathway ontologies to functionally annotate genes that show changes in expression and that cluster together. Second, we identify upstream modulators and regulators of the differentially expressed genes to provide a systems perspective on SES and immunity. Such a view also isolates potential targets for remediation. Finally, we consider the behavioral and health-related factors that may explain associations between SES and the immune cell transcriptome. Results reveal that SES is associated with widespread dysregulation of immunity involving intricately interrelated differentially expressed genes, transcription factors, and protein-protein regulators. Furthermore, BMI is a likely, potent mechanism driving these patterns.

## Methods and materials

### Add health and differential gene expression

The National Longitudinal Study of Adolescent to Adult Health (Add Health) is a representative study of adolescents in the Unites States who were followed into adulthood over five waves of data collection^39^. Study participants provided informed written consent with respect to all aspects of the Add Health study in accordance with the University of North Carolina School of Public Health Institution Review Board (IRB). Transcriptomic profiles of the consenting participants were collected during Wave V of the Add Health Study (2016–2017) via an intravenous blood draw (age of subjects range from 33 to 43 years). The access to restricted use Add Health transcriptomic data was obtained by completing a contractual and data use agreement. Additional detailed information on the study design, interview procedures, consent procedures, demographic assessments, collection, sequencing and quality control of the blood sample, and derivation of the analytical samples is reported in Supplementary Methods and in previous studies^40–42^. Furthermore, the data analysis and all methods presented in this work were carried out in accordance with the relevant ethical guidelines and regulations. We draw on the mRNA-seq data of 4015 subjects with complete information on the models’ variables. Socioeconomic status composite scores were calculated using the sum of standardized indicators of education, income, occupation, and subjective socioeconomic status of the early adult subjects^42–44^.

Genes with low counts were excluded from the analysis (see Supplementary Methods). After normalizing the raw mRNA-seq counts using a weighted trimmed mean of log expression ratios (TMM normalization)^45^ using the *edgeR*^46^ package in R, we analyzed genes whose expression varied significantly by the early adulthood socioeconomic composite score using a linear model analysis^47,48^. We controlled for covariates that could influence mRNA abundance levels: sex, self-described race, age, pregnancy status, sample analysis plate, number of hours fasting prior to blood sample collection, use of anti-inflammatory medication (e.g., NSAIDS, COX-2 inhibitors, inhaled corticosteroids), instances of common subclinical symptoms (e.g., colds, flu), and common infectious or inflammatory diseases (e.g., infection, allergies) in the 4 weeks prior to blood sample collection. We also corrected for batch effects using the *ComBat* function in the *sva* package^49^ in R.

Our overall analytic strategy is to (1) estimate clusters of genes across the whole genome and, within these clusters, identify genes that differentially expressed (DE) by SES (hereafter, SES–DEG); (2) characterize the biological function of these DE genes and gene clusters that are likely to have DE genes; (3) identify transcription factors and their protein neighbors that are associated with these DE genes and act as a networked system; (4) determine the relative functional relevance of SES–DEG and upstream regulators in manifesting immune dysregulation, and, finally, (5) identify behavioral mediators that may account for associations between SES and DE genes and their upstream regulators.

### Whole-genome clusters and cluster-SES relationship

Processed gene expression data from 14,251 transcripts in 4015 individuals were subject to unsupervised clustering using Weighted Gene Coexpression Network Analysis (WGCNA)^50^. We identified a total of 19 clusters and the number of genes in each cluster and the clusters’ overlaps with the SES–DEG are shown in Supplementary Fig. S1. To identify the clusters that have a significant relationship to SES, we modelled the cluster eigengenes (a summarized expression vector of each cluster) as a linear function of SES as in the differential expression analysis. Additionally, we performed a Fisher exact test to identify clusters that show an enrichment for SES–DEG. Together, the two tests resulted in clusters that, (1) have a significant cluster-SES relationship and (2) are enriched for SES—up or downregulated genes (see Supplementary Fig. S2). Four clusters (Cluster 7, 11, 13 and 17) had eigengenes that are significantly differentially expressed by SES. Of the 4 clusters, Cluster 11 showed an overrepresentation for SES—downregulated genes, while Clusters 7, 13 and 17 showed overrepresentation of by SES—upregulated genes. In this context, upregulation refers to a positive association between SES and mRNA abundance levels.

### Functional enrichment analysis of the differentially expressed genes and significant clusters

Functional enrichment analysis for the SES–DEG (see Supplementary Fig. S3 and Supplementary Dataset S1) and WGCNA identified cluster genes (see Supplementary Fig. S4 and Supplementary Dataset S2) was performed using R Bioconductor package *ReactomePA*^51^ to identify the biological function of the genes (FDR *p* < 0.05). The Reactome results are organized in a hierarchical structure of biological pathways with each biological pathway being a node that shows parent–child relationships^52^. We relied on this parent–child relational database to pool together multiple pathways under the same parent node in order to better understand the large-scale changes (up to 3 hierarchical levels). The significance of the parent node was determined by its most significant child.

Functional enrichment analysis of the SES–DEG and the upstream regulators was performed using the ClueGO^53^ plugin in Cytoscape^54^. This plugin allows for the combined analysis of multiple gene lists using a preselected ontology. We analyzed the SES–DEG (up- and downregulated gene lists) along with their upstream regulators (transcription factors and protein neighbors) with the Reactome ontology.

### Identifying key controllers of genes exhibiting differential expression by SES

Upstream regulators of the SES–DEG (Set A; see Supplementary Fig. S5) were categorized into (1) transcription factors that are themselves differentially expressed (Set B; see Supplementary Fig. S5), (2) protein neighbors of the differentially expressed transcription factors (Set C; see Supplementary Fig. S5), and (3) transcription factors that putatively modulate the expression of differentially expressed genes (Set D; see Supplementary Fig. S5). Marbach et al.^33^ constructed tissue-specific regulatory networks that linked transcription factors and genes with a score based on a curated collection of sequence binding motifs. Those transcription factors that had a medium or greater confidence (> 0.4) of modulating the expression of the differentially expressed genes in blood tissue were included in the set of upstream regulators (Sets B and D). A total of 643 transcription factors were identified in the blood tissue-specific gene regulatory network. 304 transcription factors had gene interactions with at least a medium confidence score. Protein neighbors of differentially regulated transcription factors were obtained using the STRING database^55^. Each protein-protein interaction (PPI) in STRING is annotated with a score that indicates the confidence of the interaction. Only neighbors with scores of at least high confidence (> 0.7) were included in the set of upstream regulators (Set C). Thus, Set A represents the DE genes and Sets B, C and D together constitute their upstream regulators. The 304 tissue-specific transcription factors had interactions with 8543 unique protein-protein neighbors. 1750 neighbors exceeded the confidence threshold for PPI.

### Possible mediators of SES and DE genes and upstream regulators

We examined behavioral and psychobiological process that might mediate associations between Wave V early adulthood SES and the expression of the genes and upstream regulators using a counterfactual mediational framework^56^. The mediators included Body Mass Index (BMI), perceived stress (based on Cohen’s Perceived Stress Scale^57^), current self-reported smoking status, consumption of alcoholic drinks (days drank over past 30 days; categorized as 0 drinks, 1–2 drinks, 3–5 drinks, and more than 5 drinks per occasion), financial stress (self-reported difficulty in paying bills), and access to health insurance. We also compared the mediation of BMI with the mediation observed for waist circumference, which has been reported to be a more accurate measure of fatness^58^.

### Randomization test of differentially expressed genes and upstream regulators

We quantified the statistical significance of the observed results by performing randomization tests based on 1000 randomly generated sets of differentially expressed genes. Random samples were drawn from the entire genome to obtain a set of genes equal in number to Set A (see Supplementary Fig. S5). Sets B, C, and D were derived from every randomly generated Set A using the same procedure used with the SES–DEG. We then computed the significance (empirical *p*-value) of every actual gene in Set A (DE genes) and Sets B, C, and D (upstream regulators)^59^ by comparing them to their respective sets from the 1000 randomly generated sets. We obtained *p*-values for each of the sets of genes by combining the *p*-values of every gene in the set using Fisher’s method.

## Results

### Transcriptional alterations with SES are characterized by organism wide dysregulation

**Figure 1:**
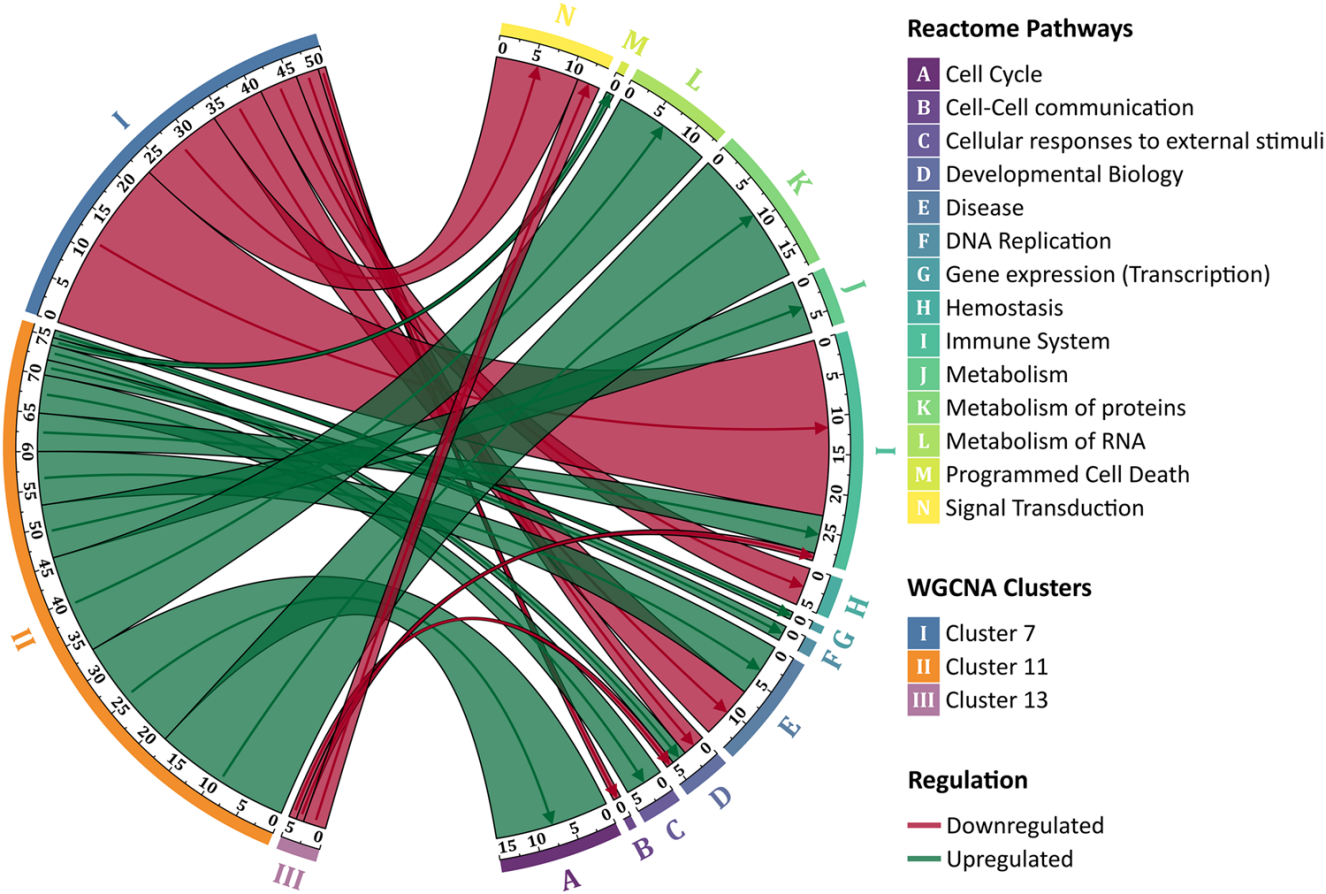
Enrichment analysis of the WGCNA clusters exhibiting significant cluster-SES relationships. Eigengenes from individual clusters were identified using WGCNA and then examined for significant associations with SES (adjusted *p* < 0.05). Enriched biological pathways for significant clusters (adjusted *p* < 0.05) were then examined using Reactome. Here, upregulation refers to a significant positive association between SES and expression. The chord diagram shows the connection between the clusters (on the left half of the chord diagram) and the enriched grouped pathways (right). The width of the arcs and the lines connecting WGCNA identifies clusters to the enriched pathways correspond to the number of pathways that are dysregulated. For example, Cluster 13 (identified as “III” on the left half of the chord diagram) significantly downregulates 5 pathways. 1 pathway each in Developmental Biology and Immune system (“D” and “I” on the right half of the figure) respectively, and, 3 pathways in Signal Transduction (marked “N”).

We performed a differential gene expression analysis followed by an enrichment analysis of the resulting SES–DEG (see Supplementary Dataset S2 and Supplementary Fig. S3). Upregulated genes indicate a significant association between high SES and high expression (i.e., a positive association). Functional enrichment of the SES–DEG (423 upregulated genes and 389 downregulated genes) showed a majority upregulated for pathways involving metabolism, signal transduction and cellular response to stress by a core of ribosomal and translational genes. Interestingly, these cytosolic ribosomal genes (*RPL*- and *RPS*-genes) were found to be downregulated with aging in an analysis of the human peripheral blood and previously linked with SES^42^. Indeed, a combined WGCNA and SES—differential expression analysis (see Fig. 1) showed a tight clustering of the ribosomal and transcriptional activity genes (Cluster 11 in Fig. 1) that are responsible for the SES-upregulated pathways. One cluster of SES-DEGs (Cluster 7) displayed dysregulation in immune system and response, hemostasis and cell death that were predominantly driven by downregulated genes, while another cluster (Cluster 11), largely comprising upregulated ribosomal genes, affected transcriptional events in several cellular functions. Cluster 13 consisted of genes involved in cell division and cell cycle control dysregulating a relatively small number of pathways in signal transduction and immune system, while Cluster 17 comprised too few genes for a meaningful enrichment interpretation.

**Figure 2:**
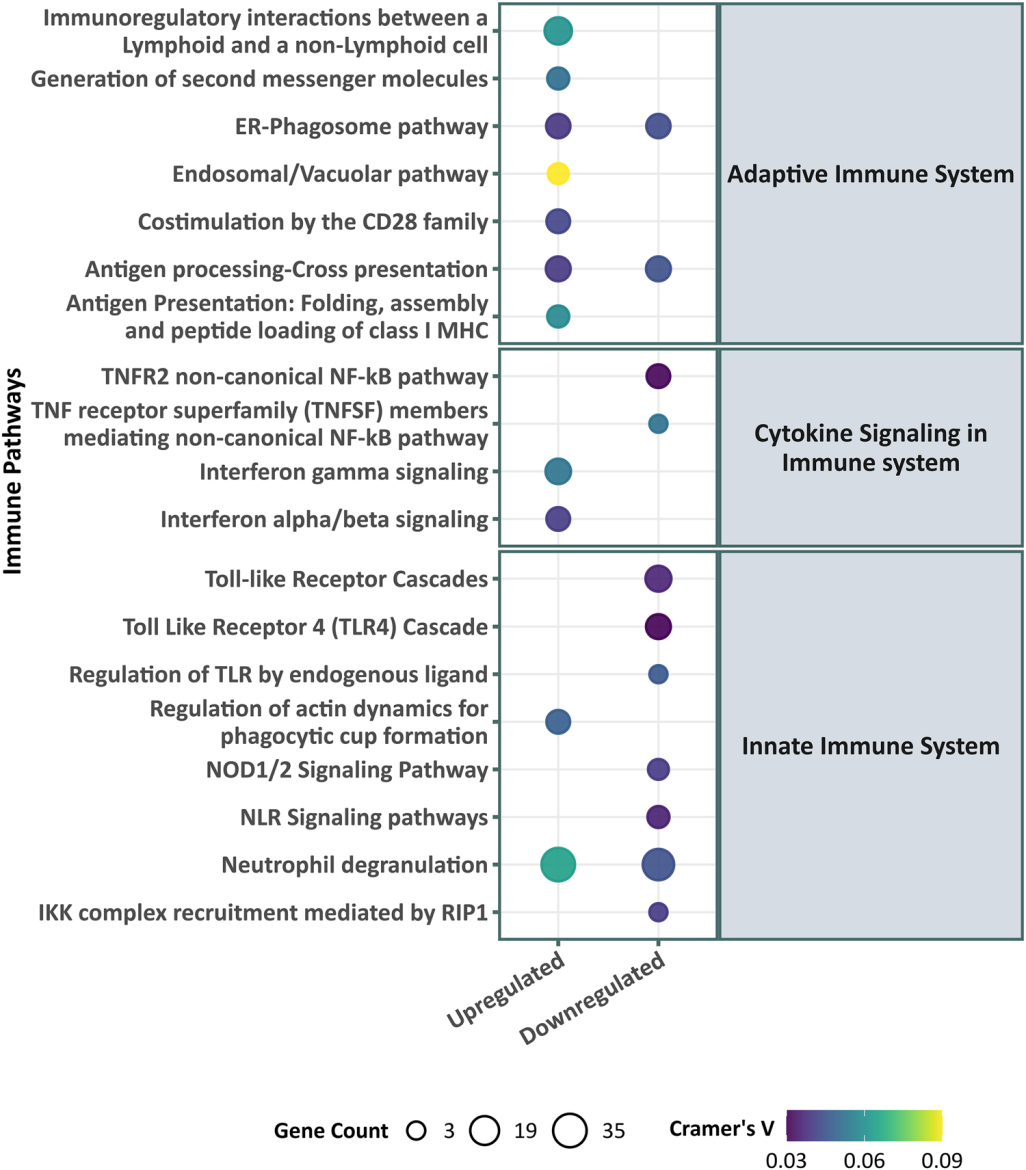
Immune pathway enrichment of the differentially expressed genes by early adult SES. Significantly enriched Reactome pathways (with immune parent nodes reported to the right, child nodes to the left) for SES–DEG. The size of the circle signifies the number of genes that contribute to the significant enrichment in a pathway and the color of the circle indicates Cramer’s V, a measure of the magnitude of association. The overrepresentation analysis shows a predominantly upregulated antiviral immune response (Interferon signaling and neutrophil degranulation) and downregulation in proinflammatory pathways (NF-κB and toll-like receptor cascade) with high SES.

An inspection of enriched pathways reveals that SES-upregulated pathways include interferon innate immune response and neutrophil degranulation (see Fig. 2). Curiously, type II interferon (IFN-γ) signaling is also upregulated. Despite the lack of direct evidence for their involvement, the genes that are tied to the upregulation of type I and type II interferon signaling do share *HLA*-genes that regulate the antiviral immune response, which may explain the overrepresentation of both classes of immunity. Figure 2 also shows an attenuation of proinflammatory pathways with higher SES (i.e., upregulation of proinflammatory pathways with low SES) via pathways in the proinflammatory nuclear factor kappa-light-chain-enhancer of activated B cells (NF-κB) and proinflammatory toll-like receptors (TLR).

### Upstream regulators structure immune dysregulation with socioeconomic disparities

**Figure 3:**
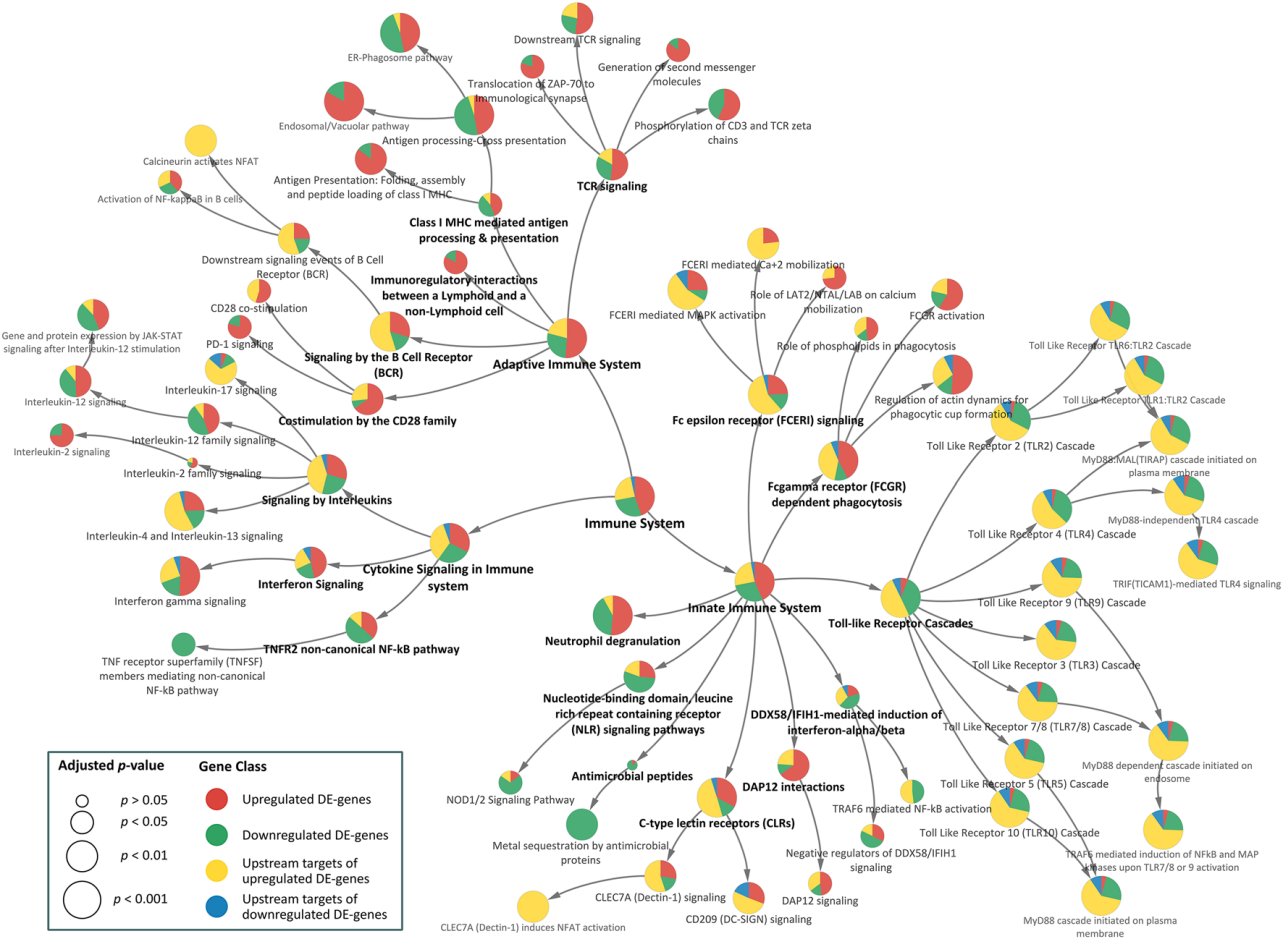
Combined functional immune enrichment analysis of the SES–DEG and their upstream regulators. Reactome pathways in the immune system are represented here as circular filled nodes. Reactome pathways are hierarchical, and the arrow connects a parent node to its child. The significance of pathways (adjusted *p* < 0.05) for the combined analysis are computed using ClueGO^53^ and represented by the size of the circular nodes. These nodes are filled with a pie chart which indicates the contribution of individual gene class. The size of the circular node signifies the adjusted *p*-value (larger nodes are more significant).

The biological pathways and molecular mechanisms associated with socioeconomic disparity were also examined via an analysis of upstream regulators (transcriptional factors and protein partners) of the DE genes. A combined functional enrichment analysis of the resulting upstream regulators (239 upstream regulators of SES—upregulated genes and 87 upstream regulators of SES—downregulated genes) and SES–DEG (see Fig. 3 and Supplementary Dataset S3) indicates a significant role of the upstream regulators in structuring the overrepresented pathways in the immune system. The upstream regulators include tissue-specific transcription factors that are (1) differentially expressed (Set B, see Supplementary Fig. S5 and Supplementary Table S1), (2) potentially regulating the expression of a differentially expressed gene (Set D, see Supplementary Fig. S5 and Supplementary Table S1), and (3) PPI neighbors of differentially expressed transcription factors (Set C, see Supplementary Fig. S5 and Supplementary Table S1). Figure 3 indicates that the immune pathways that are enriched have a larger proportion of upstream targets. Importantly, many of these pathways were also enriched by SES–DEG signifying that the patterns of immune dysregulation observed in Fig. 3 do not reflect the inclusion of upstream regulators per se, but rather reflect the significant mechanistic role played by these transcription factors and protein partners. Furthermore, the immune dysregulation observed in functional enrichment analysis of the upstream targets without the inclusion of SES–DEG (see Supplementary Fig. S6) mirror those enriched by SES–DEG alone and SES–DEG with upstream targets, suggesting that SES–DEG are not wholly responsible for SES-associated immune dysregulation.

**Figure 4:**
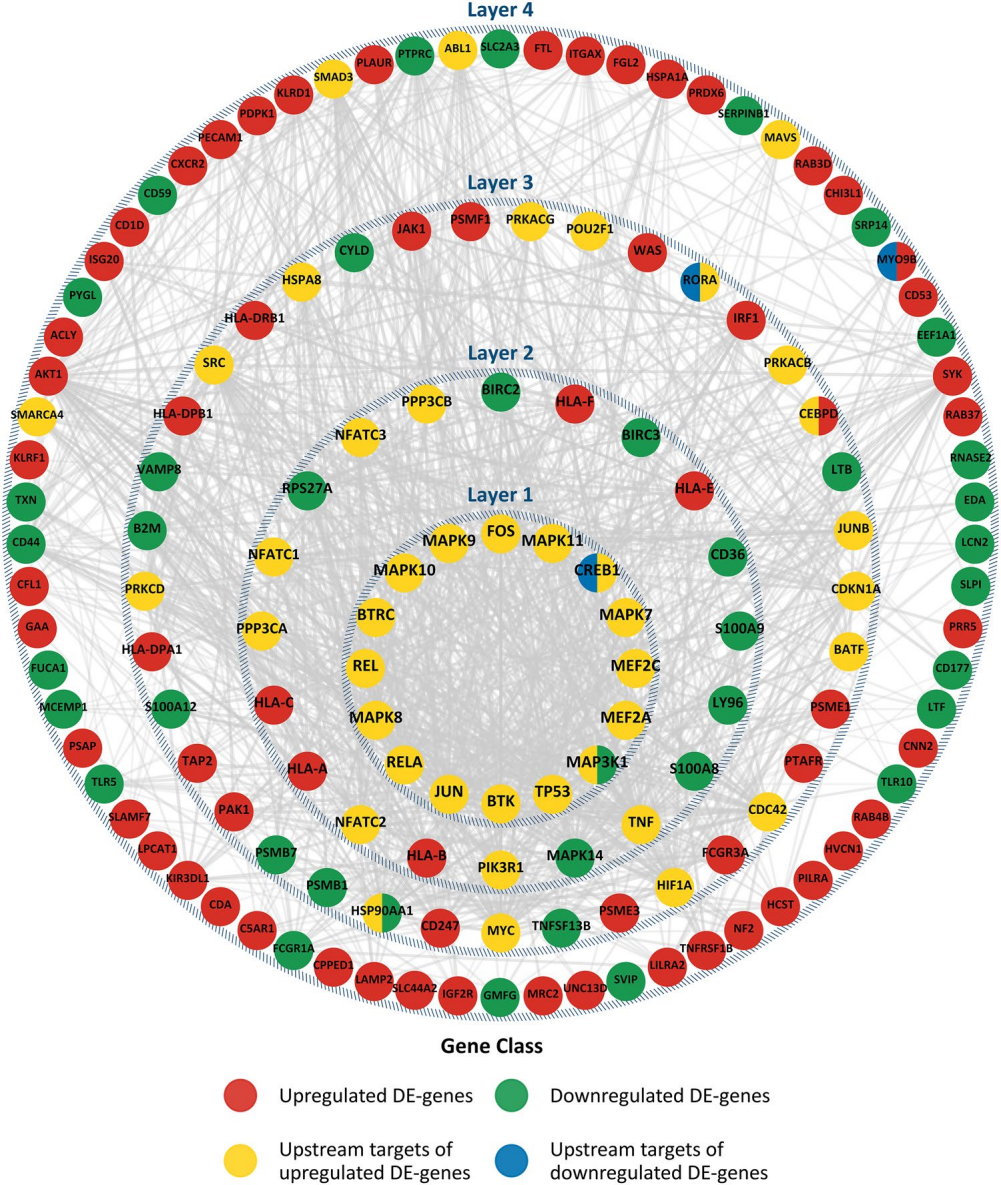
Relative importance of SES–DEG and upstream regulators to the immune dysregulation. The figure shows the upstream regulators along with the SES–DEG represented as layers (4 in total). The innermost layer represents genes and upstream regulators that are involved in the enrichment of more than 10 functional pathways (in Fig. 3) and thus they are pivotal in immune dysregulation, whereas the outermost layer consists of genes and upstream regulators that are only involved in 1 enriched biological process.

Figure 4 shows the upstream regulators along with the SES–DEG represented as layers (4 in total, where the innermost circle of genes and upstream regulators is labelled “Layer 1”, sequentially to the outermost group of genes and regulators labelled “Layer 4”) based on their interaction scores derived from the STRING database and the number of times each gene or upstream regulator is involved in functional immune pathways that are enriched in Fig. 3. The genes that are responsible for the enrichment of each immune pathway were identified. The innermost layer in Fig. 4 depicts genes and upstream regulators that are involved in more than ten functional pathways, whereas the outermost layer consists of genes and upstream regulators that are only involved in one enriched biological process. The most essential regulators involved in the dysregulation of the immune system related to SES disparities thus occupy the center of the diagram. Significantly, variations in early adult SES are prominently linked to alterations in cytokine signaling in the immune system involving interleukin and interferon gamma signaling, Toll-like receptor signaling cascade, and TNF pathways (Fig. 3 and innermost layer in Fig. 4).

Proteins most deeply linked to SES inequalities invariably revolve around the cyclic 3ʹ–5ʹ adenosine monophosphate response element-binding protein (CREB) and NF-κB pathway signaling. Variations in the activity of CREB and NF-κB selectively upregulate the transcription of interferon response factor family while simultaneously inhibiting the activity of proinflammatory interleukins in subjects with high SES. Genes and transcription factors (via the upstream analysis) that are central to the functional response to SES disparities in functional gene regulation (shown in Fig. 3) are also shown in Fig. 4. Molecules are placed in layers depending on their contribution (instances of enrichment of a pathway) to the functional immune enrichment. The inner most layer consists of transcriptional factors such as *CREB1*, *TP53*, *RELA*, *REL*, *BTRC*, *BTK* and the *MAPK-*family. *CREB* and *REL* proteins, among other important functions, play a crucial role in the activation of the fight-or-flight signaling pathways that is directly responsible in eliciting the CTRA gene expression profiles. Although a large fraction of the proteins is derived from the set of upregulated genes, it is noteworthy that these regulators can have far reaching impact and they are not always in the expected direction. This is particularly true for the proinflammatory toll-like receptors (TLR) (see Fig. 3) pathways, which have a larger proportion of downregulated genes than upregulated genes. However, they also functionally interact with the upstream regulators connected to upregulated genes. The greater influence of the upstream regulators connected to upregulated genes suggests that the downregulation of certain genes could transpire as a consequence of inhibitory activity of the transcription factors.

### Social/behavioral mediators of SES-transcriptome associations

**Figure 5:**
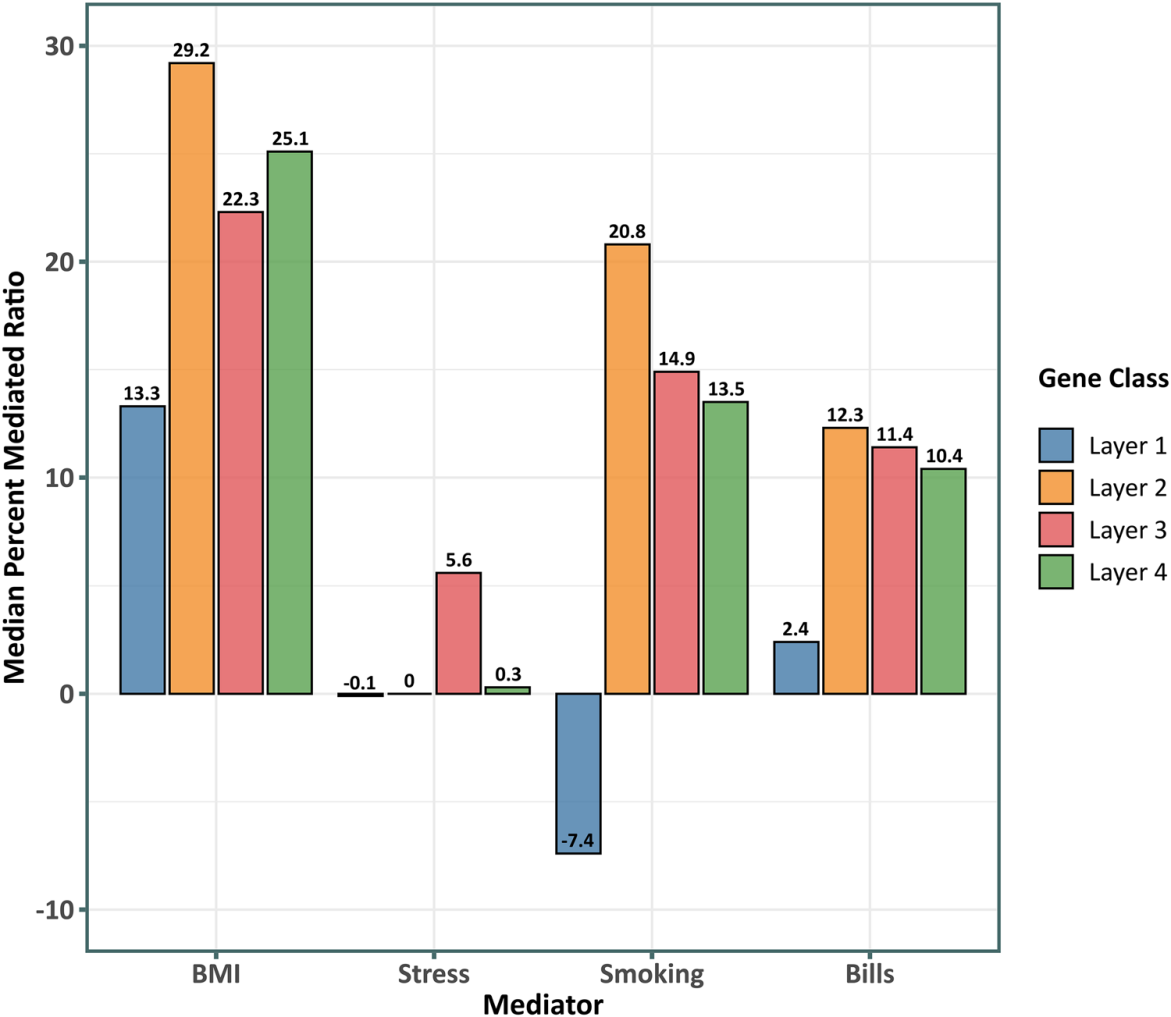
Mediation analysis for common behavioral risk factors and the layers of upstream regulators of SES-associated immune system dysfunction. The median percent mediated ratio (Average casual mediated effect (ACME)/total effect) is shown for mediational models for the risk factors and the layers of SES–DEG and upstream regulators in Fig. 4. All the mediational models were significant (aggregated adjusted *p*-value < 0.05) (also see Supplementary Figs. S6 and S7). Negative ratios suggest a pattern of suppression.

Figure 5 reports the median percentage mediated ratio for key SES-related social/behavioral processes in every layer of SES-related gene regulation. Intriguingly, the inner most group of genes that are most centrally implicated in SES-associated dysregulation, are also mediated the least (lowest median percentage mediated ratio) by every behavioral risk factor. However, this could reflect the fact that the inner most group of genes are not themselves differentially expressed despite potentially inducing larger mediated changes in the outer layers of genes. BMI presented the strongest explanation of the association between the transcriptional response of the gene groups and SES, followed by smoking tobacco (also see Supplementary Figs. S7 and S8). No significant mediation was observed for financial stress or access to health insurance. Furthermore, we observed no significant differences between BMI and waist circumference in mediating the SES—associated immune transcriptome (see Supplementary Fig. S9).

## Discussions

The present analyses expand the scope of prior studies of SES-related alteration in transcriptomic profiles of human immune response genes by identifying new genomic functional impacts (e.g., ribosomal biology) and new features of the gene regulatory architecture of SES (e.g., *TP53*, *BTRC*, *BTK* and *MAPK* transcriptional control pathways). Consistent with prior research, we find that SES is negatively associated with pro-inflammatory pathways in the nuclear factor kappa-light-chain-enhancer of activated B cells (NF-κB) and proinflammatory toll-like receptors (TLR). Our findings also link high SES to elevated type II interferon (IFN-γ) signaling and identify a related upregulation of *HLA*- genes, which may underpin both type I and type II interferon effects.

The analyses extend previous research by mapping networks of upstream blood-specific transcriptional factors and protein interactions that could play a vital role in structuring the observed transcriptional landscape. The complexity of the impact of socioeconomic inequalities on the immune system and its association with diseases with widely varying pathologies warrants a systems-oriented approach to comprehensively analyze the SES-related perturbations in the immune transcriptome. To our knowledge, most prior research on SES focuses solely on transcriptomic alterations with a particular focus on pro-inflammatory action^30^. Here, we include the upstream regulators to depict an enhanced view of dysregulation with SES, shedding light on a tightly knit group of transcription factors that play a central role in modulating the transcriptomic alterations.

Our findings map a central network of upstream regulators that vary as a function of early adult SES (central positions in Fig. 4). Given the lack of change in the expression of the genes that encode these transcriptional factors, receptor-mediated post-transcriptional modification of these transcriptional factors (e.g., receptor-mediated phosphorylation of *CREB1*, *TP53*, *RELA*, *REL*, *BTRC*, *BTK* and *MAPK*) might modulate the expression of their downstream targets.

Despite the congruence between our results and the CTRA model in terms of dysregulated pathways, the findings show that SES disparities in early adulthood, interestingly, do not alter the same set of genes identified in previous studies of CTRA. For example, the attenuated interferon innate responses in the CTRA model is possible through the direct suppression of *INFA* and *INFB*. Here, we observed upregulated *HLA-*genes that additionally regulate the antiviral innate response. These findings call for a for further study in SES-modulated genes in the immune system beyond the signaling pathways already implicated in structuring the conserved transcriptional response to adversity (CTRA) RNA profile^60^.

The observed SES-related transcriptional perturbations are associated with both up- and downregulated functional pathways in the immune system. Social stress-induced gene alterations in humans are associated with diseases that include both upregulation of the immune system as well as suppressed immune responsiveness with lowered social status. This seemingly perplexing pattern has been explained by the selective characteristics of the immune transcriptome, i.e., the increase in expression of certain pro-inflammatory genes and the repression of groups of antiviral immune response genes^40,61–63^. It is, therefore, unsurprising that a similar pattern of dysregulated immune pathways is emergent in the blood transcriptomic landscape of subjects in Add Health with contributions of enrichment from both up- and downregulated gene clusters. Such a finding calls for additional research that moves beyond the initial general finding that stressors upregulate proinflammatory genes and downregulate antiviral genes.

Lastly, among the common mediating (or possible explanatory) mechanisms studied here, BMI consistently emerged as a plausible mediator of the SES associations with immune cell gene regulation. Smoking also appears to play a significant role in the SES-related transcriptional alterations. These results underscore the importance of gene regulatory network approach in formulating a comprehensive understanding of psychosocial stressors and their impact on biological mechanisms early in life. Studies have already established that changes caused by socioeconomic disparities in early adulthood could have far-reaching implications for chronic conditions in later adulthood. Identification of novel regulators of such perturbations is an important step in formulating a mitigating strategy.

We used tissue-specific regulatory networks to link transcription factors to differentially expressed genes, and subsequently the STRING database to find protein interaction partners. There were 643 transcription factors identified in the gene regulatory network, with an even a smaller number (304) having a confidence score that is above the threshold used (0.4). High confidence (threshold > 0.7) protein-protein neighbors derived from the whole network of 643 transcription factors numbered 3051 (18,875 total protein-protein neighbors), of which 1750 were pruned for the 304 transcription factors. Given the central role of many of the transcription factors and protein partners, one could argue that the set of upstream regulators found from the SES–DEG could be equal to a set of upstream regulators derived from a random set of genes. To examine this possibility, we performed randomized trials by starting with randomly selected sets of “DE” genes to then derive these upstream regulators of the random sets. We subsequently compared the upstream regulators of each random set of “DE” genes to our observed results (see Supplementary Figs. S10 and S11). While some of the individual transcription factors may not reach statistical significance (Supplementary Fig. S10), the entire set of the upstream regulators is highly significant (Supplementary Fig. S11).

## Limitations

Several limitations are noteworthy. First, the hypotheses and the subsequent results are driven by the mRNA abundance data collected once from every participating subject. The repeated collection of transcriptomic data would be essential to address their highly transient nature, which is likely associated with considerable noise. Second, the identification of upstream transcriptional regulators of gene expression is performed with the aid of tissue-specific gene regulatory networks. These networks link genes to transcription factors based on experimental evidence and assign a confidence score to every identified transcription factor. It is, therefore, possible that transcription factors that play a central role in cell maintenance and cell cycle may be implicated without having a substantive role in the etiology or progression of dysfunction. We tried to account for such effects using a randomization experiment. However, direct measurements of protein abundance are required to concretely determine the role of every transcription factor. In the absence of proteomic assay data, inferring transcription factor abundance from publicly available chromatin immunoprecipitation followed by sequencing (ChIP-seq) data sources that have similar gene expression alterations as those observed with lowered SES, could offer valuable insights and presents an important extension of the current work. Finally, because the design is not experimental, the findings cannot be interpreted as casual relationships. This limitation is especially salient for the casual identification of social and behavioral mediators of SES-related immune dysregulation. Future research could usefully examine them and other mediators in a casual framework to disentangle expected tissue repair response to stress induced by these risk factors (e.g., obesity, smoking) and global immune system dysfunction.

Nevertheless, results suggest that a network of transcription factors and protein partners play a pivotal role in modulating the SES-related transcriptional response that precipitates dysregulated immune system response in terms of inflammation and interferon innate immunity. These central actors are important targets for future research connecting health disparities and socioeconomic inequalities. The results highlight the need for system-oriented analyses to comprehensively map the biological impact of SES disparities and they represent an essential step forward in identifying targets for possible prevention and intervention.

### Supplementary Information

Supplementary Information 1.

Supplementary Information 2.

Supplementary Information 3.

Supplementary Information 4.

## Data Availability

Add Health data are available at https://www.cpc.unc.edu/projects/addhealth/documentation/. All the data used in these analyses, except for the transcriptomic data are not restricted. The mRNA-seq data is available via a restricted data contract. Additional information and application for the restricted-use data can be accessed through the Carolina Population Center (CPC) data portal at https://data.cpc.unc.edu/projects/2/view. The Cytoscape sessions, supplemental datasets and R codes used in these analyses are available at https://github.com/socialgnome/Immune-SES.
