# Supplementary material for: Socioeconomic inequalities in early adulthood disrupt the immune transcriptomic landscape via upstream regulators
Source: Sci Rep. 2024 Jan 13;14:1255. doi: 10.1038/s41598-024-51517-6 (PMC10787749; doi:10.1038/s41598-024-51517-6)
Supplement: Supplementary file 4 — Supplementary Information 4. [file 41598_2024_51517_MOESM4_ESM.pdf]

SUPPLEMENTARY INFORMATION

FOR

Socioeconomic inequalities in early adulthood disrupt the immune transcriptomic landscape via upstream regulators

Sudharshan Ravi [a*], Michael J. Shanahan [a,b], Brandt Levitt [c],

Kathleen Mullan Harris [c,d], Steven W. Cole [e]

[a] Jacobs Center for Productive Youth Development, University of Zürich, Zürich, CH 8050

[b] Department of Sociology, University of Zürich, Zürich, CH 8050

[c] Carolina Population Center, University of North Carolina at Chapel Hill, Chapel Hill, NC 27516

[d] Department of Sociology, University of North Carolina at Chapel Hill, Chapel Hill, NC 27599-3210

[e] School of Medicine, University of California, Los Angeles, Los Angeles, CA 90095

* Corresponding author

Sudharshan Ravi

Jacobs Center for Productive Youth Development

University of Zürich

Andreasstrasse 15

Zurich, CH - 8050

sudharshan.ravi@jacobscenter.uzh.ch

Table of Contents

A. Datasets

Dataset S1: SES – DEG enriched reactome pathways.

Dataset S2: Enriched reactome pathways for the genes in the WGCNA-identified clusters.

Dataset S3: SES – DEG and upstream regulators enriched reactome pathways in the immune system.

B. Supplementary Methods

Study Protocol: Collection, Sequencing, Quality Control and Raw Data Processing

C. Supplementary Tables

Table S1: Size of SES – DEG and upstream regulators.

D. Supplementary Figures

Figure S1: Number of genes in the WGCNA-identified clusters that are differentially expressed for early adult SES.

Figure S2: WGCNA Clusters that show a significant cluster-SES relationship and enriched for SES – DEG.

Figure S3: Functional enrichment analysis of SES – DEG.

Figure S4: Functional enrichment analysis of the genes in WGCNA-identified clusters.

Figure S5: An overview of the workflow to identify upstream regulators of the SES – DEG.

Figure S6: Functional immune enrichment analysis of the upstream regulators of SES – DEG.

Figure S7: Mediation models for common behavioral risk factors and SES – DEG and upstream regulators.

Figure S8: Mediation models for common behavioral risk factors and the layers of upstream regulators of SES-associated immune system dysfunction.

Figure S9: Mediation models for BMI and waist circumference and SES – DEG and upstream regulators.

Figure S10: Probability distribution of the differentially expressed genes and upstream regulators in the randomized trials.

Figure S11: Cumulative probability of the differentially expressed genes and upstream regulators in the randomized trails.

Supplementary Methods

Study Protocol: Collection, Sequencing, Quality Control and Raw Data Processing

Introduction

The National Longitudinal Study of Adolescent to Adult Health (Add Health) is an ongoing, nationally-representative, longitudinal study of the social, behavioral, and biological linkages in health and developmental trajectories from early adolescence into adulthood. The cohort was drawn from a probability sample of 132 middle and high schools and is representative of American adolescents in grades 7-12 in 1994-1995. The adolescent cohort has been followed for 25 years with in-home interviews in 1995 (Wave I), 1996 (Wave II), 2001-02 (Wave III), 2008 (Wave IV), and 2016-2018 (Wave V). Wave V was conducted when respondents are aged 32-42. The study design included an embedded genetic sample of pairs of identical twins, fraternal twins, full siblings, half siblings, and adolescents who grew up in the same household but have no biological relationship. The study population is a multiracial and multiethnic sample with substantial numbers of individuals with Hispanic and Asian ancestry. Additional information about the design of Add Health can be found in previous studies [1,2].

Sample Collection

The study's sample refers to randomly selected participants in Add Health who consented to provide a blood specimen for RNA analysis during a physical examination visit. All Wave V respondents who participated in the Wave V home biomarker collection visit (home visit) and agreed to a blood draw were eligible to provide a PAXgene sample. We use data from tranches 1, 2, and 3 (approximately one year apart) which were collected in 2016-17 (n=4,543).

As part of the Wave V survey, respondents were asked if they consented to a home visit by Add Health's data collection partner company (EMSI between 2016-2017 or Hooper Holmes between 2018-2019). If they consented, they were contacted by a field examiner (FE) from the partner company to schedule a date, time and location for the FE to meet them. The vast majority of home visits occurred in the respondent's home. All FEs were certified phlebotomists with at least three years of phlebotomy experience. They were also required to have completed a customized training program for the Wave V biomarker collection protocols.

During the home visit, a venous blood draw was performed for those respondents who agreed to it (94%). Venous blood was collected using a bar-coded room temperature PAXgene™ Blood RNA tube designed to draw 2.5 ml of blood into the BD Hemogard™-capped, plastic tubes (BD, Franklin Lakes, NJ). Typically, 2.5 ml PAXgene RNA tubes yield 1,000-8,000 ng of total RNA. Transcriptome profiling by Illumina HT12 bead arrays consumed approximately 360 ng of sample, and the remaining 900-7,900 ng of RNA was returned to Laboratory for clinical Biochemistry Research at University of Vermont

(LCBR) for long-term archival storage. Each PAXgene RNA tube contained a proprietary reagent that lyses cells and stabilizes intracellular RNA. Complete blood draws took about 10 seconds and were followed immediately by inversion 8 to 10 times. Samples were then shipped within 24 hours of collection by FedEx to the LCBR. On arrival, samples were scanned into the laboratory data management system and stored at -80°C. Samples have been shown to retain integrity for up to 2 freeze-thaw cycles and 50 months at -80°C. 5,000 specimens were shipped in three batches over a 36-month data collection period to the UCLA Social Genomics Core Laboratory for sequencing analysis.

Collection of Other Relevant Data

Clinical covariates

Survey questions were asked at the time of blood collection to address potential clinical confounders. Such data included respondents' compliance with physicians' requests to avoid caffeinated beverages and foods, heavy physical activity, smoking, and alcohol intake preceding biomarker measurements; time since last ingestion of food; pregnancy, recent infection, and other conditions under which measurements were made.

Medication inventory

A medication inventory was also generated to record potential pharmacological confounders. Data on medications were crucial because many therapeutic classes of medications affect gene expression. Collection of medication data in Wave V closely followed the efforts at Wave IV in which a general internist and cardiovascular epidemiologist with expertise in medication classification manually assigned therapeutic classes using two on-line coding databases (Lexi-Comp® On-LineTM, Lexi-Comp®, Inc.; Hudson, OH and MICROMEDEX® 1.0, Thomson Reuters Healthcare, Inc; Philadelphia, PA).

Transcriptome Sequencing

Genome-wide mRNA expression profiles were assayed in 360 ng of 30 ng/µl total RNA. RNA was extracted under RNAse-free/PCR clean BSL2 conditions using automated nucleic acid extraction systems (Qiagen QIAcube) and deposited into 96 well plates. RNA concentration and purity was assessed by spectrophotometry (Nanodrop ND1000) and RNA integrity was assessed using an Agilent Bioanalalyzer (Agilent, Palo Alto CA). Genome-wide transcriptional profiles were obtained using target cRNA synthesis (Ambion TotalPrep) and hybridization to Illumina Human HT-12 bead arrays in the UCLA Neuroscience Genomics Core Laboratory following the manufacturer's standard protocol (Illumina, San Diego CA). Importantly, cDNA synthesis was conducted using the Lexogen QuantSeq 3' FWD library preparation chemistry which amplified only the final 65 nucleotides of each transcript. Resulting sequencing reads were separated by sample based upon barcode, paired with their mate, filtered for Phred quality scores below Q30, and adaptors were trimmed using trimmomatic. Reads were then mapped to a reference genome Hg38 using Star aligner 2.6. Further information on the methodology is reported in previous studies [3].

Sample Quality

RNA concentration

RNA was extracted from whole blood as described above. Concentrations of extracted RNA ranged from 6.70 ng/µl to 142 ng/µl for year 1, 4.40 ng/µl to 266 ng/µl for year 2, and 0.17 ng/µl to 60.54 ng/µl for year 3. Median RNA concentrations for year 1 was 36.50 ng/µl, for year 2 was 33.50 ng/µl, and for year 3 was 25.13 ng/µl. For samples with concentrations less than 30 ng/µl, RNA was concentrated using Qiagen minelute columns. This accounts for 142 samples in year 1, 492 in year 2, and 1,613 in year 3. Previous literature has shown successful RNA sequencing has been conducted with samples as low as 0.250 ng/µl [4]. No filter was used to censor samples with low RNA concentration.

RNA purity

RNA purity can be assessed by measuring the ratio of optical absorbance at 260 nm to optical absorbance at 280 nm. At the 260 nm wavelength, nucleic acids absorb light while the aromatic amino acid side chains of proteins absorb light at 280 nm. The ratio of the two is an indication of how much protein is contaminating the RNA sample. Illumina, the manufacturer of the sequencing instrument used, recommends a ratio of 260/280 of 1.7 or higher for samples to be sequenced. No filter was applied based upon RNA purity.

RNA integrity

RNA integrity is assessed by examining the ratio of 28S ribosomal RNA to 18S ribosomal RNA. Ribosomal RNA comprises over 80% of RNA in the cell and can be divided into these two sizes (2 kb and 5 kb) depending upon whether it is derived from the small or large subunits. There is a theoretical expectation that the abundance of 28S ribosomal RNA will be greater than 2 times the abundance of 18S ribosomal RNA. Deviation from this ratio can be largely explained by degradation of the larger species of ribosomal RNA. Because messenger RNA is closer in size to the larger 28S species, degradation of 28S ribosomal RNA is an indication that there is degradation of messenger RNA. Literature values of this ratio span from 0.5 to 2.5 [5]. In year 1, the minimum, median and maximum 28S/18S ratios are 0.2, 1.5 and 3.7. In year 2, the minimum, median and maximum 28S/18S ratios are 0.2, 1.5 and 2.8. In year 3, these values were not reported. A filter of 28S/18S = 0.5 was applied to censor samples with low RNA integrity.

RNA quality

The Agilent tapestation instrument measures RNA integrity and quality using a numerical score of 1-10 called the RNA integrity number (RIN). This measure uses an electrophoretic approach to determine the homogeneity of the size of the RNA in the sample. This measure frequently is used to augment information provided by the 28S/18S ratio because the previous measure determines degradation while this measure describes the homogeneity of remaining RNA after degradation. Illumina recommends only running samples with RIN scores of greater than 7. In year 1, the minimum, median and maximum RIN scores were 3.1, 7.8 and 9.2. In year 2, the minimum, median and maximum RIN scores were 3.1, 8 and 9.3. In year 3, the minimum, median and maximum RIN scores were 1.1,

8.1, and 10. RIN scores below the Illumina threshold account for 119 participants in year 1, 146 participants in year 2, and 6 participants in year 3. A filter of RIN=3 was applied to censor samples with low RNA quality.

Full analysis on sample quality is available as a technical bulletin, Add Health Project, Carolina Population Center, University of North Carolina at Chapel Hill.

Reads

Number of reads

The number of reads produced from the transcriptomic sequencing is highly dependent upon the instrument used and the sample quality. Deviations from median numbers of reads for any given sample may be an indication that the reads arising from that sample are skewed in some way either due to an amplification jackpot event or preferential amplification of certain sequences (kmer content). Alternatively, low numbers of reads arising from a sample may be due to poor sample quality or sample contamination. It is therefore important to identify participants whose sample produced unusual numbers of reads. In year 1, the minimum, median and maximum numbers of reads produced were 0.15, 12.33 and 30.85 * 10^6 reads. In year 2, the minimum, median and maximum numbers of reads produced were 0.13, 12.75 and 50.19 * 10^6 reads. In year 3, the minimum, median and maximum numbers of reads produced were 0.86, 13.51, and 800.60 * 10^6 reads. Typically, outliers are pruned from the data set to avoid the potential introduction of questionable data. In year 1, there was 1 sample with $<10^6$ reads and no other extreme outliers. In year 2, there were 4 samples with $<10^6$ reads. In year 3, there were 1 sample with $<10^6$ reads. A filter of 1 * 10^6 reads was applied to censor samples producing unexpectedly low numbers of reads.

Mapping of reads

The number of reads that map to genes is of utmost importance to determine if certain samples should be excluded. The percent of reads failing to map at all, mapping to multiple genes or mapping to a single gene were calculated. In year 1, the minimum, median and maximum percentages of reads mapping were 67.4%, 95.20% and 98.7%. In year 2, the minimum, median and maximum percentages of reads mapping were 8.95%, 95.9% and 98.45%. In year 3, the minimum, median and maximum percentages of reads mapping were 74.68%, 98.80% and 99.40%. The same measures for uniquely mapping reads were 48.50%, 75.6% and 87.3% for year 1; 6.86%, 75.1% and 90.24% for year 2; and 40.72%, 78.96%, and 91.11% for year 3. The discrepancy of mapped reads compared to uniquely mapped reads can be explained by reads that mapped to multiple sites. The minimum, median and maximum percentage of reads mapping to multiple genes were 10.3%, 19.1% and 32.4% for year 1; 2.09%, 19.37% and 35.79% for year 2; and 7.59%, 19.78%, and 56.28% for year 3. There was one sample in year 2 that had a mapping percent of less than 8.95%. There were 2 plates in year 2 that had unmapped percentages of reads of about 25%. Typically, 70-90% of reads map to a gene [6]. A filter of percent uniquely mapping reads < 25% was applied to censor samples that contained the majority of their reads failing to map to the human genome.

Raw Data Processing

Preprocessing filtering

RNA sequencing and subsequently mapping of reads against a reference genome produces a gene count matrix of reads for each gene in the reference genome. However, there may not be any reads or a statistically significant number of reads for any given gene. Therefore, it is common practice to filter the data set to remove genes for which there are no or few reads. To accomplish this, an R package called *Limma*, *Voom* and *EdgeR* were used.

Conversion to differential gene expression object

The raw gene count matrix for year 1, year 2, and year 3 contained 60676 genes for 1,143, 1542, and 2,110 samples, respectively. The 3 hemoglobin genes were removed for each of the two matrices and the remaining data sets were converted to differential gene expression (DGE) objects using the *EdgeR* operation '*DGEList*'. DGE objects contain the gene count matrix as well as a complete listing of the samples by identifier, their group association, library size and normalization factor.

Low read filtering

The DGE object was then filtered to remove genes with low expression. *Limma* contains an operation called '*filterByExpr*' which implements a filtering strategy to keep genes that have worthwhile counts in a minimum number of samples. Precisely, the function retains genes that have "counts per million (cpm)" above *k* in *n* samples. *k* is determined by the user defined threshold of counts and by the sample library sizes and *n* is determined by the smallest size of sample based on grouping. In our analysis, *Limma* removes any gene for which the count is less than 10 in 95% of samples or greater.

Normalization of reads

Read counts for each gene fail to account for the library size so an operation must be conducted to normalize read numbers for the individual's library size. This is done using the *Limma-Voom* R package. First, normalization factors are calculated from the DGE object '*samples*' metric using '*calcNormFactors(method='TMM')*' in the *Limma* package. This generates a normalization factor for each sample. Then the *Limma-Voom* package is used to calculate a normalized pseudocount for each gene for each sample. *Voom* retains the gene count variance for each sample and converts the expression value such that all libraries are artificially the same size and level of gene expression can be compared between libraries. The *Voom* package generates a data object that contains the DGE sample data set with updated normalization factors, an updated expression matrix, a matrix of weights calculated from the normalization factor, and a list of samples. The *Voom* expression matrix is used for further analysis.

Supplementary Tables

Supplementary Table S1

Gene/TF Count	Upregulated	Downregulated
Set A	423	389
Set B	10	4
Set C	168	44
Set D	61	39
SES - DEG	423	389
Upstream regulators	239	87

Table S1: Size of SES – DEG and upstream regulators. Number of SES – DEG and upstream regulators identified in our workflow (see **Supplementary Fig. S5**)

Supplementary Figures

Supplementary Figure S1

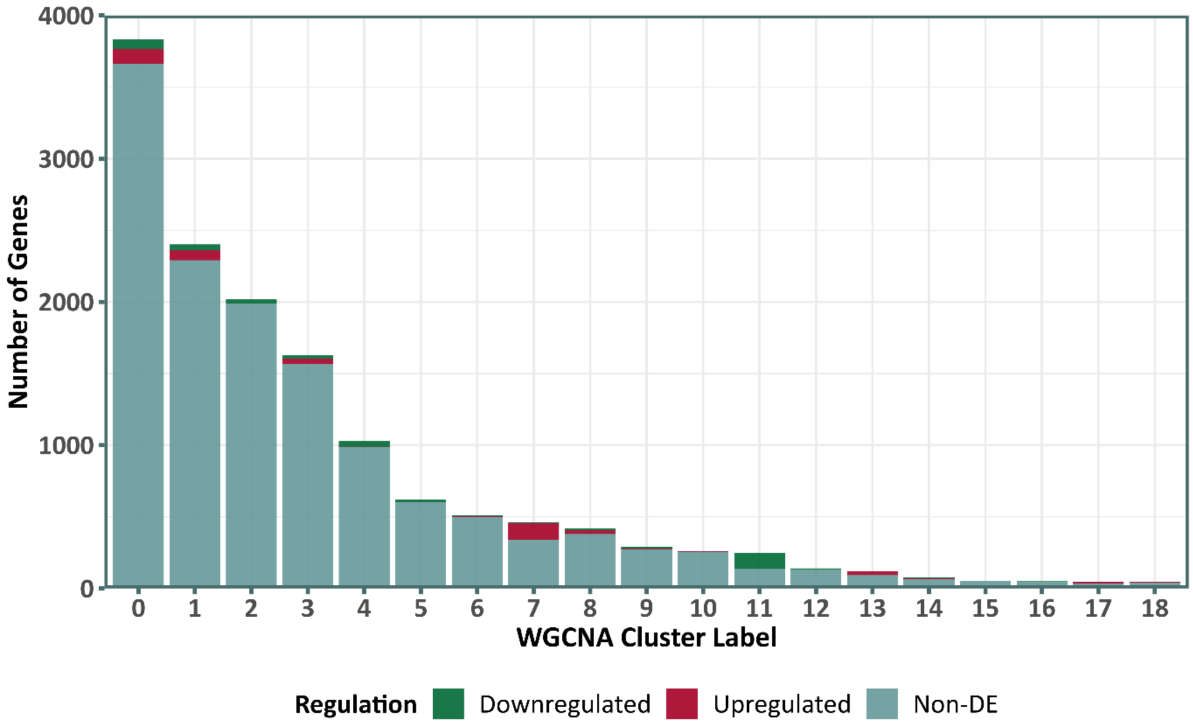

Figure S1: Number of genes in the WGCNA-identified clusters that are differentially expressed for early adult SES. Genes were clustered using WGCNA, and we identified 19 total clusters. The figure shows the number of genes in each cluster that are differentially expressed by early adult SES (i.e., ranging from low SES to high SES). Here, upregulation refers to a significant association between SES and expression (i.e., a positive association).

Supplementary Figure S2

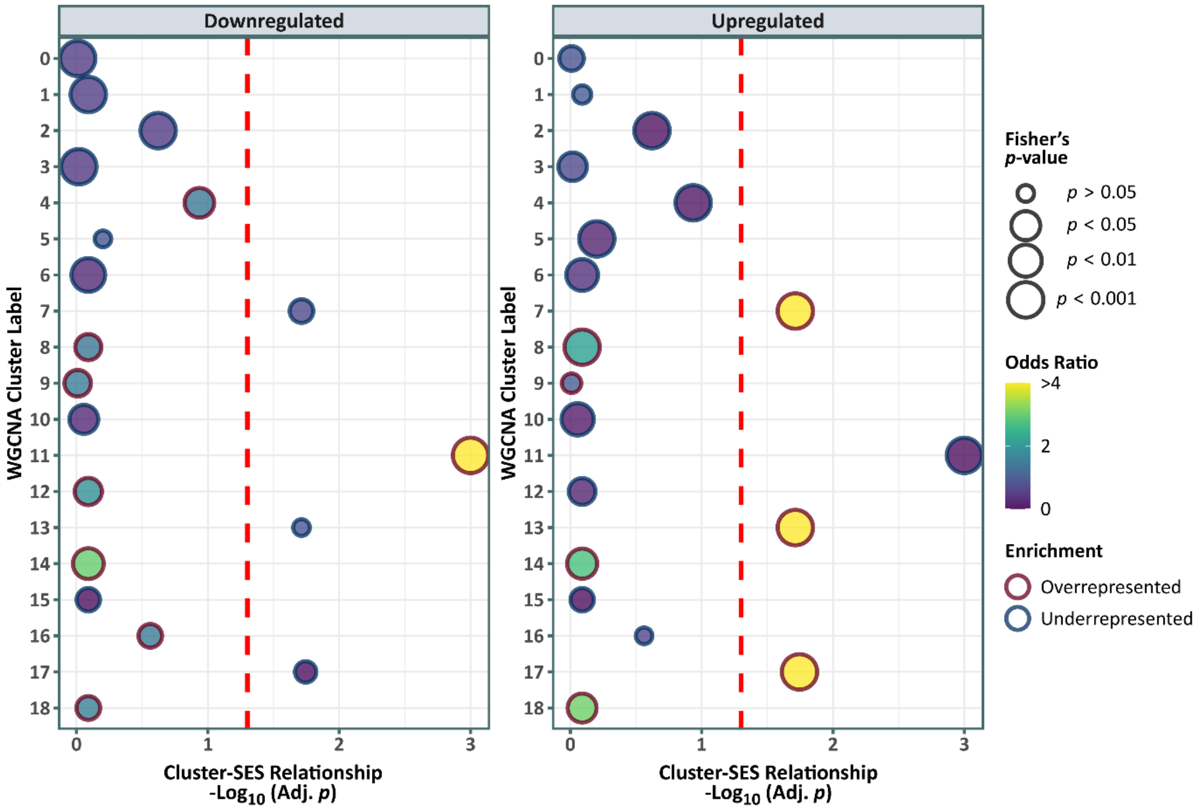

Figure S2: WGCNA Clusters that show a significant cluster-SES relationship and enriched for SES – DEG. Clusters identified by WGCNA (19 in total) were tested for significant SES dependence by modeling the cluster eigengene of each cluster as linear function of SES (similar to the differential expression analysis). The negative log of adjusted *p*-value of cluster-SES relationship is represented along the x-axis. The red dotted line represents an FDR - adjusted *p* of 0.05. Clusters 7, 11, 13 and 17 showed a significant cluster-SES relationship. The clusters were tested for enrichment of SES downregulated (left panel) and upregulated (right panel) genes using a Fisher test. The size of the bubble plot in the figure corresponds to the Fisher exact test *p* – value, while the color represents the odds ratio. Clusters with odds ratio greater than 1 (indicating an overrepresentation) are marked with a red outline.

Supplementary Figure S3

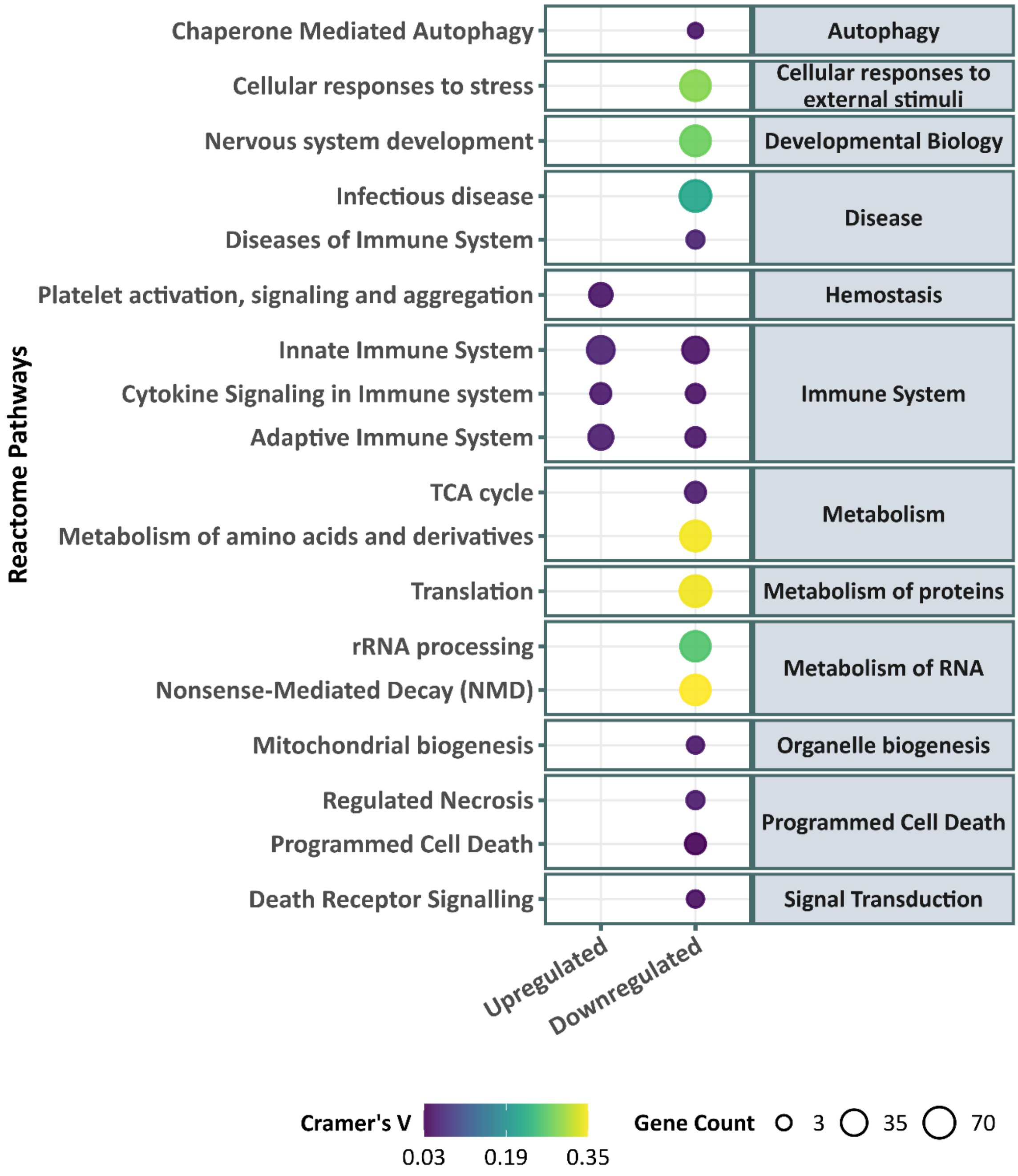

Figure S3: Functional enrichment analysis of SES – DEG. Significantly enriched Reactome pathways (adjusted $p < 0.05$) (with parent nodes reported to the right, child nodes to the left) for the SES – DEG. The size of the circle signifies the number of genes that contribute to the significant enrichment in a pathway and the color of the circle indicates Cramer's V, a measure of the magnitude of association.

Supplementary Figure S4

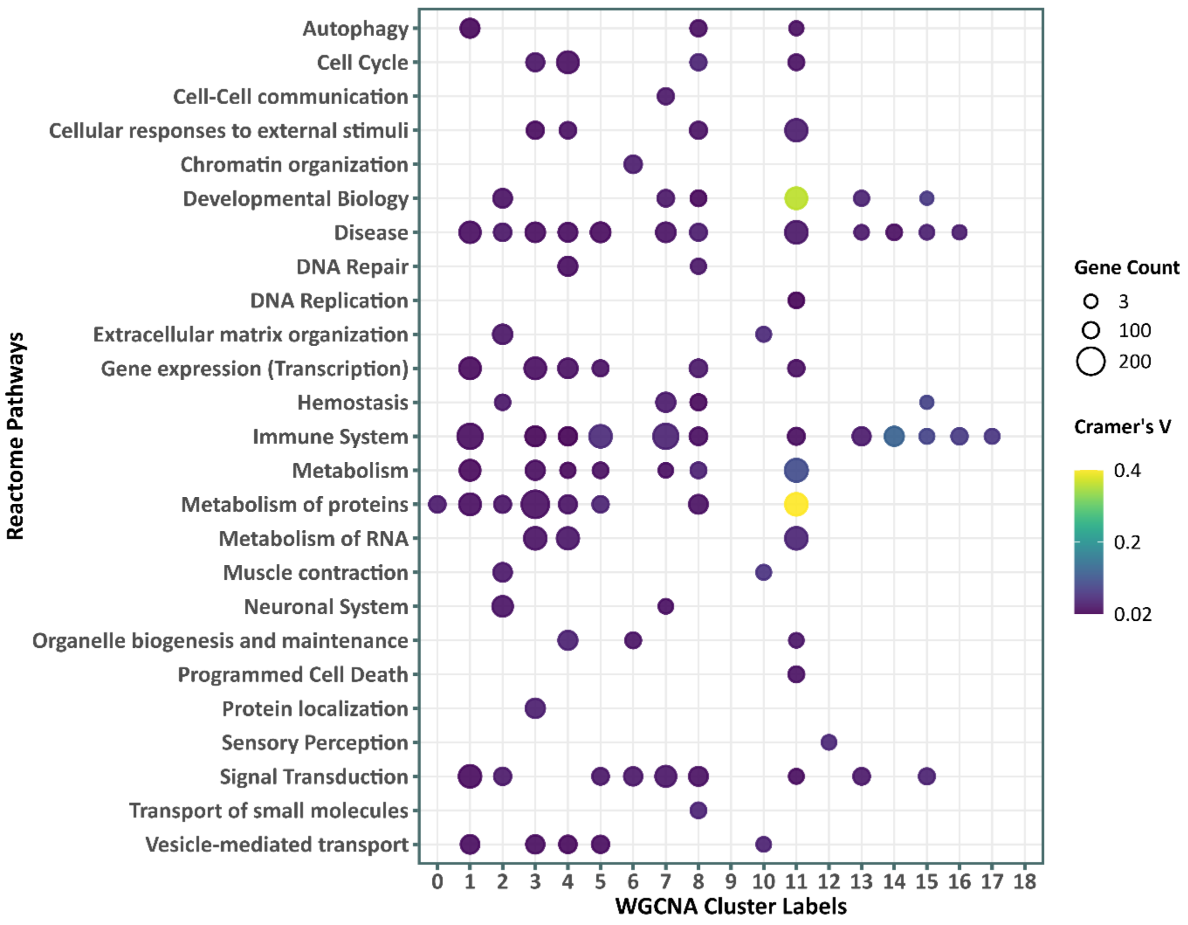

Figure S4: Functional enrichment analysis of the genes in WGCNA-identified clusters. The genes clustered using WGCNA were examined for functional enrichment using Reactome ontology. Significantly enriched parent Reactome pathways (adjusted $p < 0.05$) are represented as bubbles. The color of the nodes indicates the Cramer's V value, and the size of the nodes signify the number of the genes that contribute to the enrichment.

Supplementary Figure S5

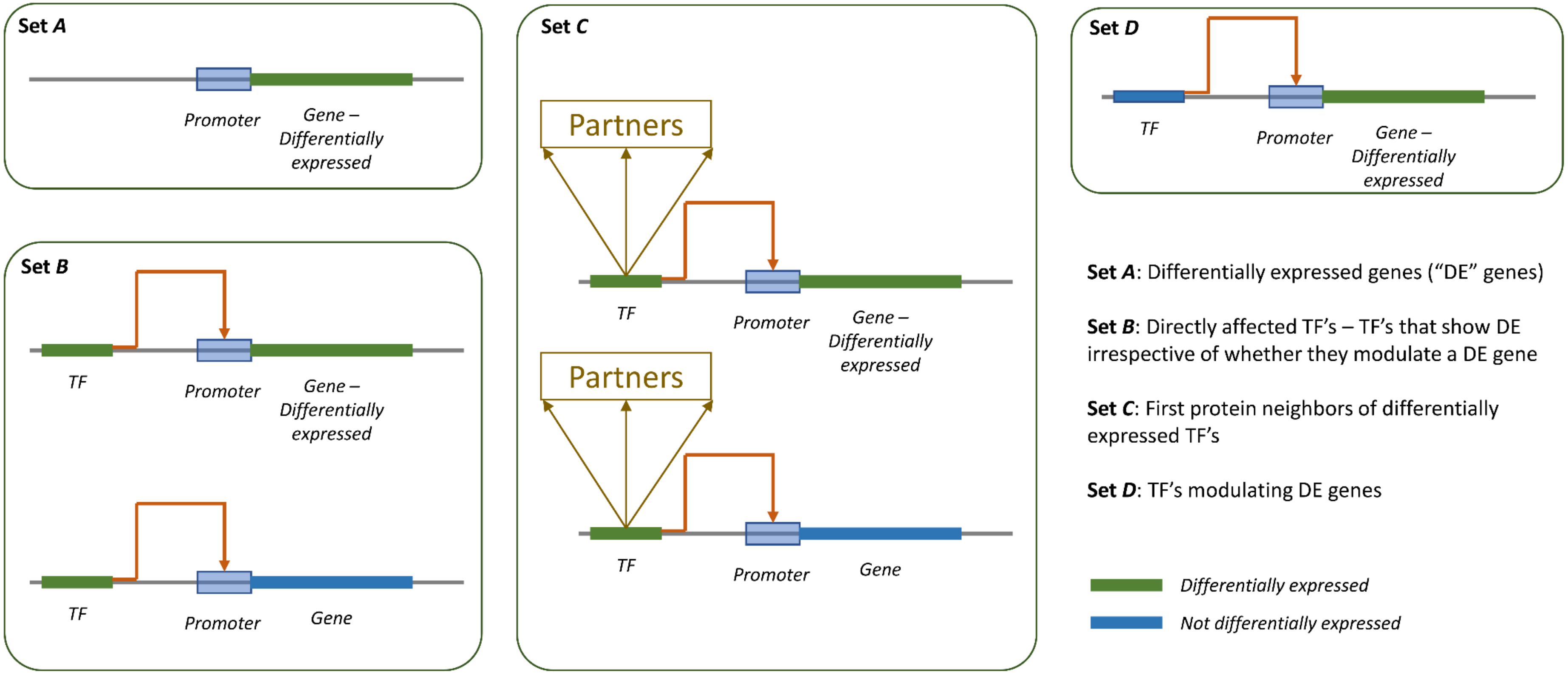

Figure S5: An overview of the workflow to identify upstream regulators of the SES – DEG. Set A represents the SES – DEG. These differentially regulated genes could also be transcription factors and constitute Set B. Set C indicates the protein interaction partners of the differentially expressed transcription factors and finally, Set D constitutes upstream transcription factors of differentially expressed genes that are themselves not differentially regulated. Sets B, C and D together constitute the upstream regulators of the DE genes (Set A).

Supplementary Figure S6

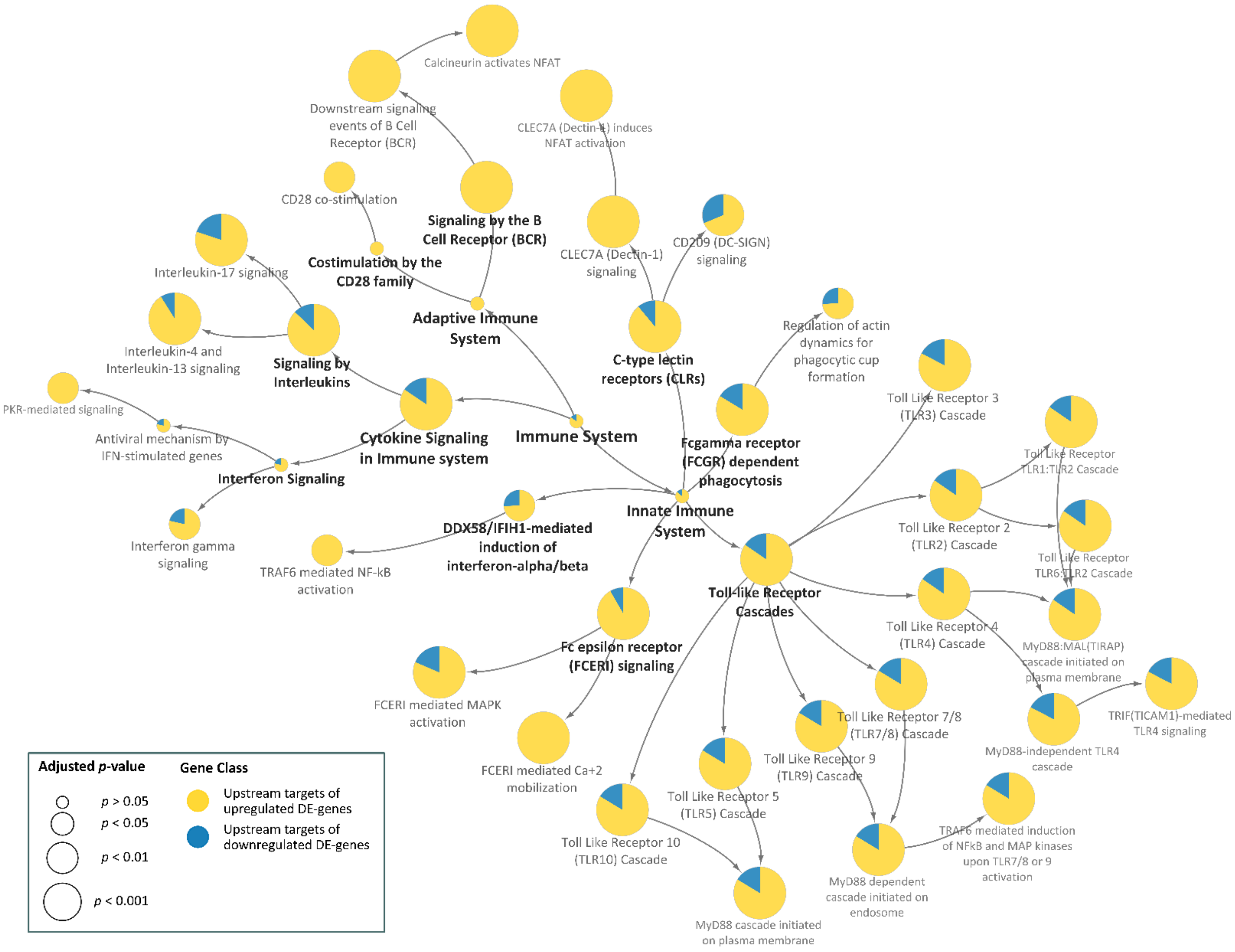

Figure S6: **Functional immune enrichment analysis of the upstream regulators of SES – DEG.** Reactome pathways in the immune system are represented here as circular filled nodes. Reactome pathways are hierarchical, and the arrow connects a parent node to its child. The significance of pathways (adjusted $p < 0.05$) for the combined analysis are computed using ClueGO [7] and represented by the size of the circular nodes. These nodes are filled with a pie chart which indicates the contribution of individual gene class. The size of the circular node signifies the adjusted *p*-value (larger nodes are more significant).

Supplementary Figure S7

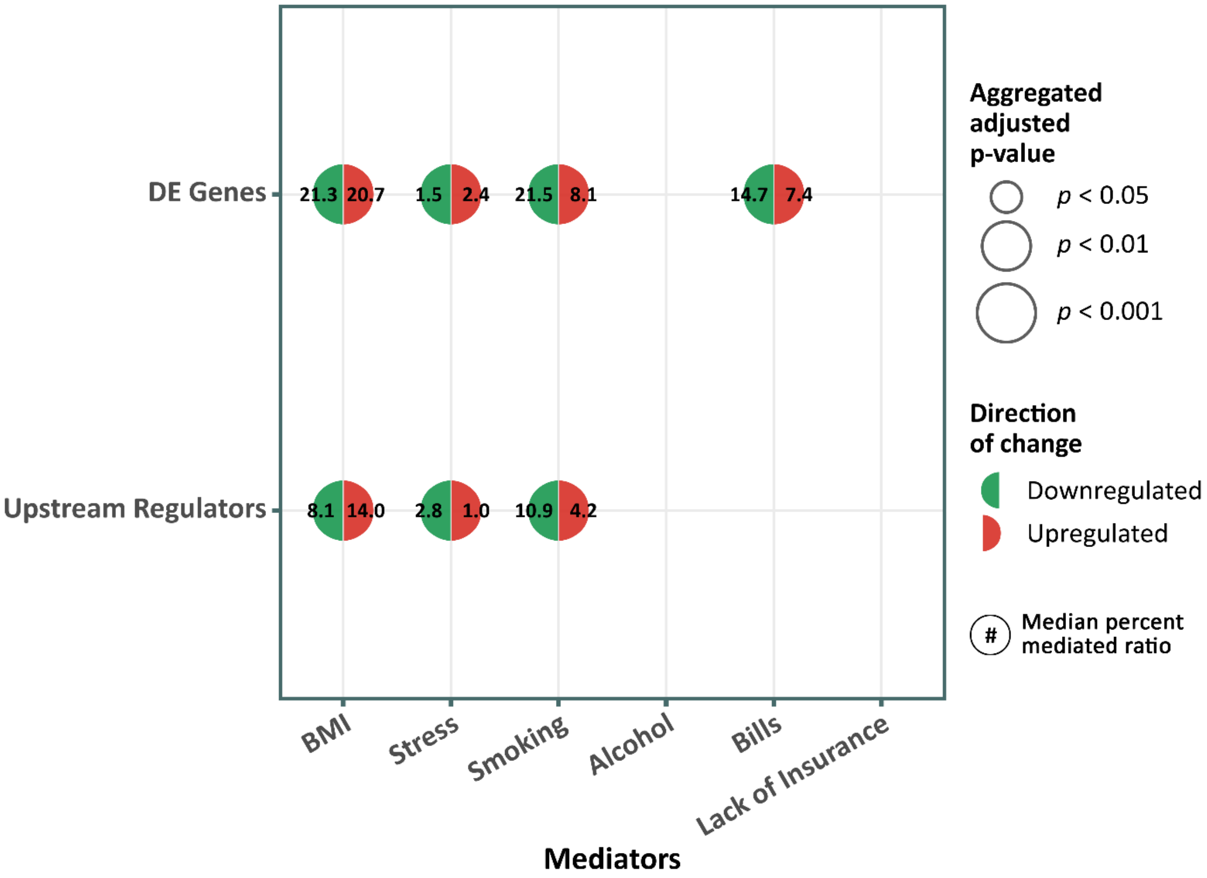

Figure S7: Mediation models for common behavioral risk factors and SES – DEG and upstream regulators. The median percent mediated ratio (average casual mediated effect (ACME)/total effect) is reported as superimposed numbers for the up- and downregulated genes and their upstream targets. The size of the half-circles corresponds to the significance of the Fisher aggregated adjusted *p* – value.

Supplementary Figure S8

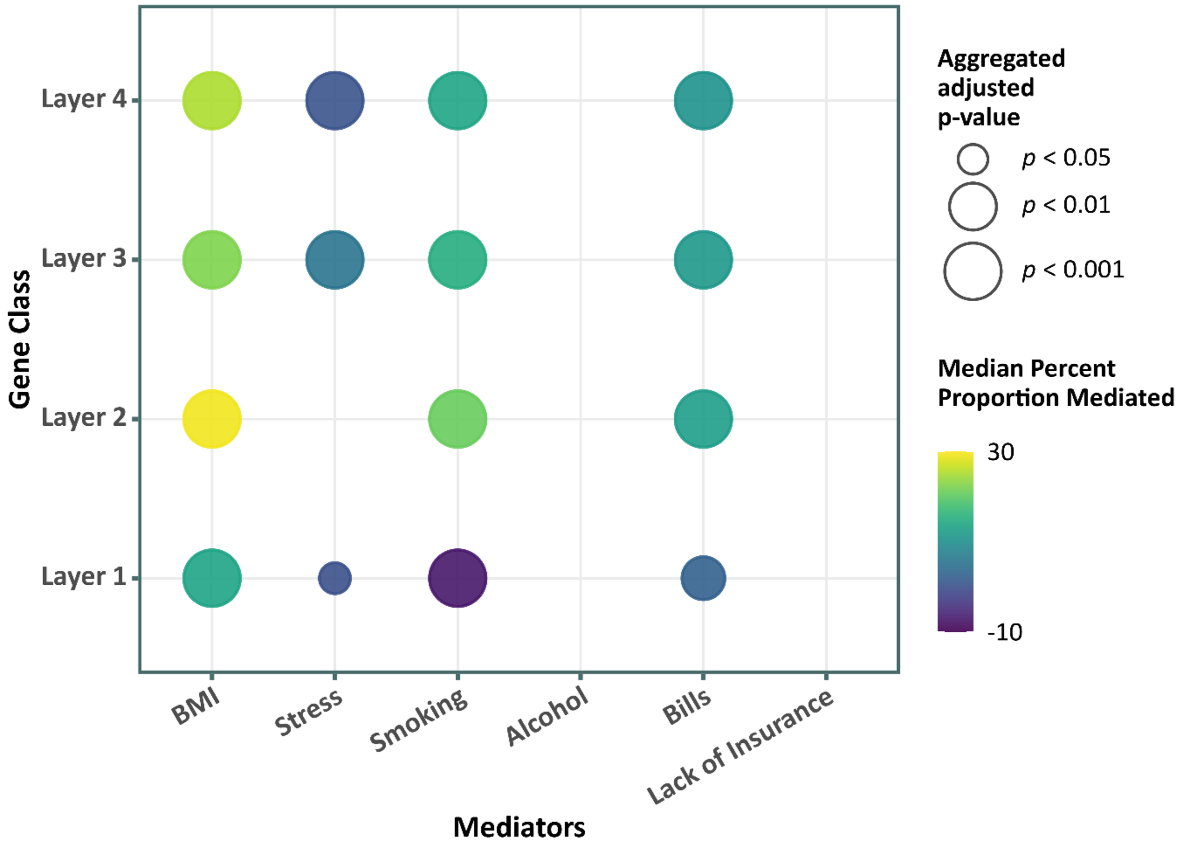

Figure S8: Mediation models for common behavioral risk factors and the layers of upstream regulators of SES-associated immune system dysfunction. The median percent mediated ratio (average casual mediated effect (ACME)/total effect) is shown in the color scale for mediational models for the risk factors and the gene classes. The size of the circle signify the Fisher aggregated adjusted p – value. Alcohol and the access to health insurance (or lack thereof) did not provide significant results. Negative values suggest a suppression effect.

Supplementary Figure S9

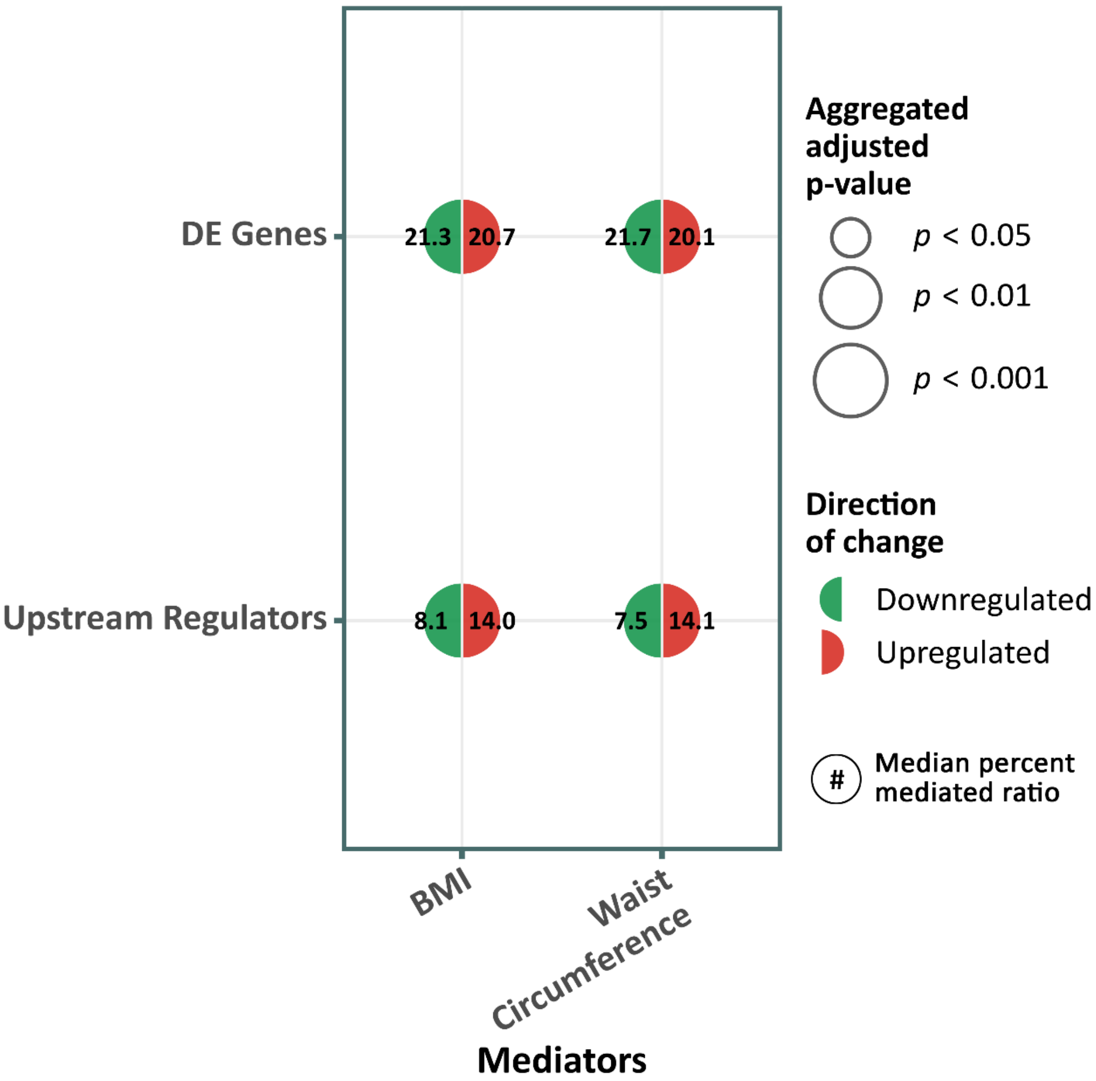

Figure S9: Mediation models for BMI and waist circumference and SES – DEG and upstream regulators. The median percent mediated ratio (average casual mediated effect (ACME)/total effect) is reported as superimposed numbers for the up- and downregulated genes and their upstream targets. The size of the half-circles corresponds to the significance of the Fisher aggregated adjusted *p* – value.

Supplementary Figure S10

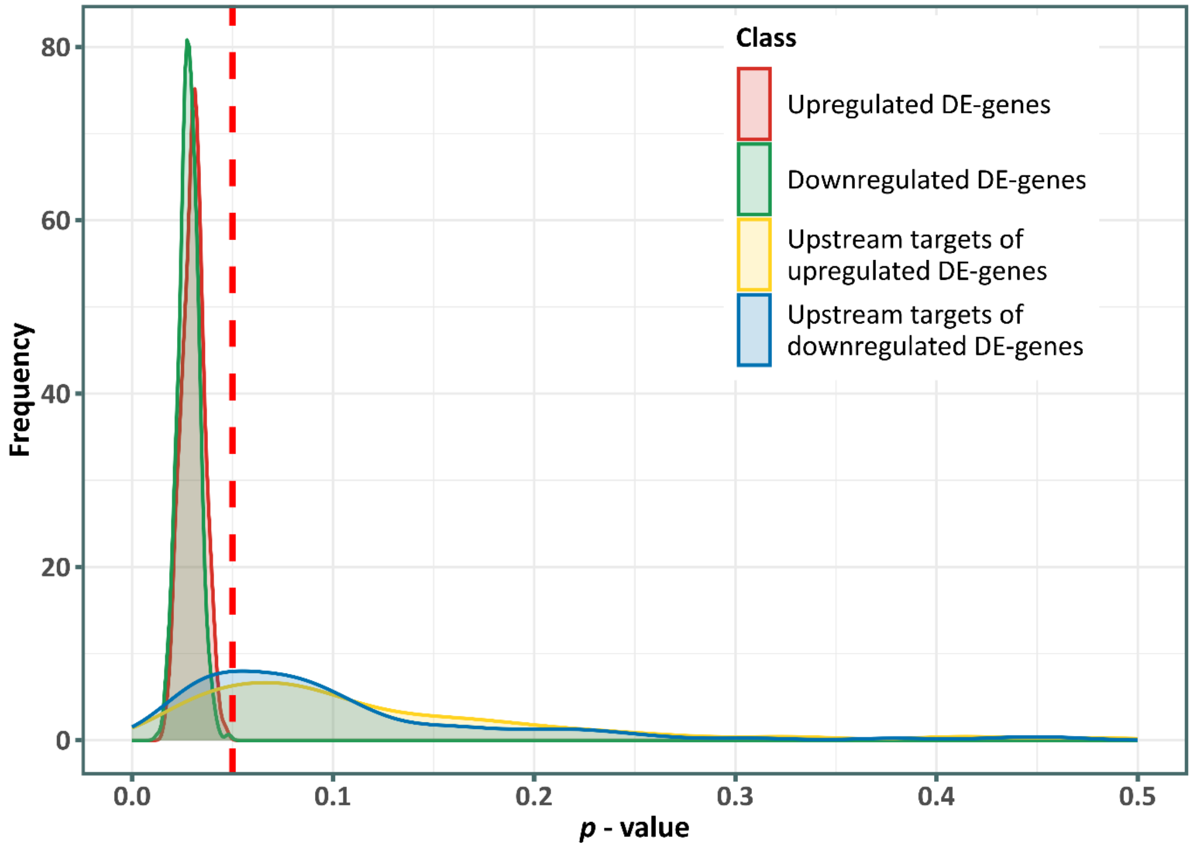

Figure S10: Probability distribution of the differentially expressed genes and upstream regulators in the randomized trials. The figure shows the density plot of the *p*-values of every actual DE gene and upstream regulator in the randomized trails. The red dotted line represents Fisher's *p* of 0.05. Although some individual upstream regulators have $p > 0.05$, the cumulative Fisher's *p* for the entire set of DE genes and upstream regulators is < 0.001 as shown in **Supplementary Fig. S11**.

Supplementary Figure S11

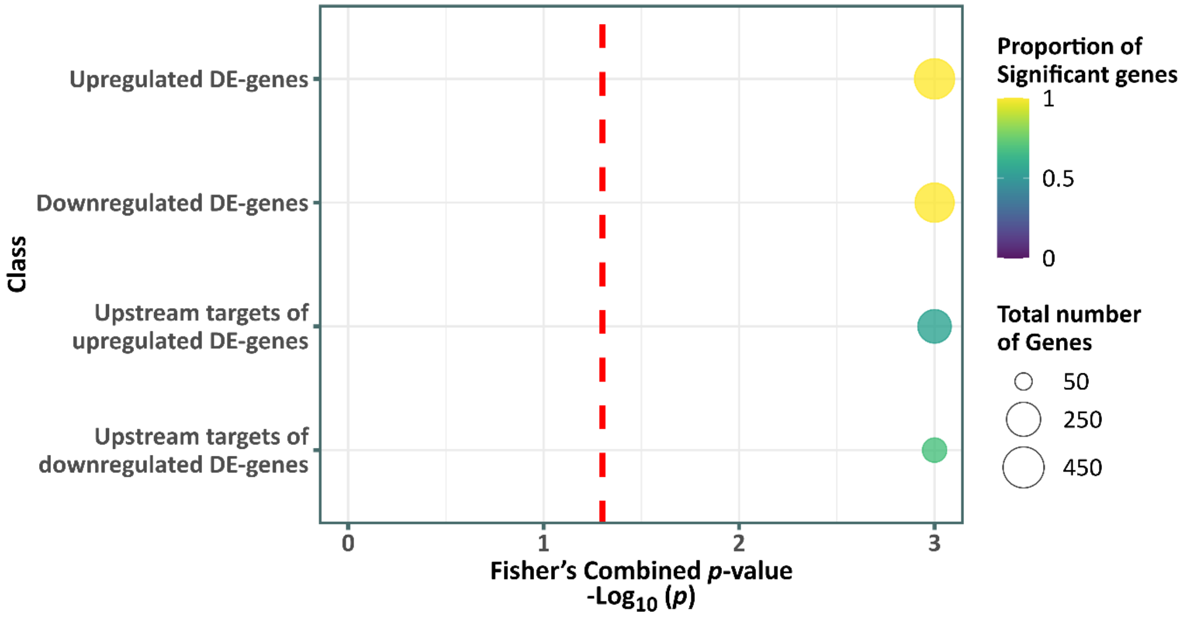

Figure S11: Cumulative probability of the differentially expressed genes and upstream regulators in the randomized trails. The Fisher's cumulative *p*-value is represented in the x-axis. The red dotted line represents Fisher's *p* of 0.05. Bubbles to the right of the dotted line signify $p<0.05$. The size of bubble plot in the figure corresponds to the total number of genes in the set, while the color corresponds to the proportion of the genes that are individually significant in the randomized trials. The cumulative probability is computed from the individual probability distributions of the actual DE genes and upstream regulators (see **Supplementary Fig. S10**). The figure shows that while some of the individual upstream transcription factors identified may not reach statistical significance, the cumulative probability of the entire set of upstream regulators is highly significant.

References

1. Harris, K. M. An integrative approach to health. *Demography* **47**, 1–22 (2010).
2. Harris, K. M. The Add Health Study: Design and Accomplishments. (2013) doi:10.17615/C6TW87.
3. Cole, S. W., Shanahan, M. J., Gaydosh, L. & Harris, K. M. Population-based RNA profiling in Add Health finds social disparities in inflammatory and antiviral gene regulation to emerge by young adulthood. *Proc Natl Acad Sci U S A* **117**, 4601–4608 (2020).
4. Sheng, Q. *et al.* Multi-perspective quality control of Illumina RNA sequencing data analysis. *Brief Funct Genomics* **16**, 194 (2017).
5. Imbeaud, S. *et al.* Towards standardization of RNA quality assessment using user-independent classifiers of microcapillary electrophoresis traces. *Nucleic Acids Res* **33**, e56 (2005).
6. Conesa, A. *et al.* A survey of best practices for RNA-seq data analysis. *Genome Biol* **17**, (2016).
7. Bindea, G. *et al.* ClueGO: a Cytoscape plug-in to decipher functionally grouped gene ontology and pathway annotation networks. *Bioinformatics* **25**, 1091 (2009).
